# Effects of Probiotic–Phytonutrient Blends on Defecation, Intestinal Barrier Function, and Gut Microbiota: A Randomized, Placebo-Controlled Trial

**DOI:** 10.3390/nu18132085

**Published:** 2026-06-25

**Authors:** Ah Young Hwang, Sunyoung Lee, JungHyun Yoon, Kyu Yeon Lee, Dong Ho Suh, Sungjae Myung, Jihye Song, Hae Jo, Dmitri Sitnikov, Jong Hoon Won, Hyun Young Park, Matthew K. Runyon, Donghyun Cho, Wilhelm H. Holzapfel, Yosep Ji, Eun Sung Jung

**Affiliations:** 1 HEM PHARMA Inc., Suwon 16229, Republic of Korea; ayhwang@hempharma.bio (A.Y.H.); jhyoon@hempharma.bio (J.Y.);; 2 Amway Research and Development, Ada, MI 49355, USA; 3 Department of Advanced Convergence, Handong Global University, Pohang 37554, Republic of Korea

**Keywords:** probiotic, phytonutrients, SCFAs, butyrate, tryptophan, intestinal barrier function, gut microbiome

## Abstract

**Background/Objectives**: Probiotic interventions are widely used to improve intestinal health; however, comparative evidence on multi-strain formulations with different potencies, particularly when combined with plant-based complexes, remains limited. This study evaluated the effects of two probiotic blends containing phytonutrients: PBP1, comprising *Lacticaseibacillus* strains, and PBP2, comprising *Lacticaseibacillus*, *Lactobacillus*, and *Bifidobacterium* strains. The effects on bowel function, microbial metabolites, and gut barrier-related markers were investigated. **Methods**: In this randomized, double-blind, placebo-controlled trial, participants received PBP1, PBP2, or placebo for 8 weeks. Stool patterns (7-day Bristol Stool Form Scale (BSFS) diary), fecal short-chain fatty acids (SCFAs), tryptophan metabolites, zonulin, and gut microbiota were assessed at baseline and Week 8. Efficacy was evaluated by comparing each intervention group with the placebo group. **Results**: Both PBP1 and PBP2 significantly increased the proportion of normal stool types (BSFS types 3–5) compared with placebo (*p* < 0.05). Fecal SCFA levels, including acetate, propionate, and butyrate, were significantly increased in both intervention groups. Notably, butyrate levels were significantly elevated compared with placebo. Fecal tryptophan levels decreased, while indole metabolites showed increasing trends, with an inverse correlation observed between tryptophan and indole, particularly in the PBP2 group. Fecal zonulin showed a decreasing trend, with significant reductions in participants with 25.0 ≤ BMI < 30.0 kg/m^2^. Microbiome analysis revealed preserved alpha diversity with selective compositional shifts, including enrichment of Lactobacillus-related taxa. **Conclusions**: Supplementation with PBP1 and PBP2 improved bowel function and was associated with changes in microbiome-derived metabolites, including SCFAs and tryptophan–indole metabolism, with BMI-dependent changes in barrier markers. These findings suggest a potential role of microbiome-mediated metabolic modulation in intestinal health.

## 1. Introduction

The human gastrointestinal tract harbors a complex and dynamic microbial ecosystem that plays a critical role in maintaining host health. A balanced gut microbiome supports digestive processes, contributes to nutrient metabolism, produces key metabolites such as short-chain fatty acids (SCFAs), and regulates immune function. In contrast, disruptions in microbial composition and impaired barrier function have been associated with altered bowel habits, including changes in stool frequency and consistency, as well as increased intestinal permeability [1,2,3,4,5]. Accordingly, modulation of the gut microbiome has emerged as a promising strategy for improving bowel function and overall intestinal health.

Probiotics are among the most widely used interventions to modulate the gut microbiome [6]. Previous studies have shown that probiotic supplementation may improve bowel function, regulate stool consistency, and support microbial balance [7,8]. However, these effects are highly strain-specific and formulation-dependent, and the underlying mechanisms remain incompletely understood [8,9]. In particular, comparative evidence on multi-strain probiotic formulations with different potencies remains limited.

Microbiome-derived metabolites represent a key mechanistic link between probiotic activity and host intestinal barrier function. Probiotic strains can modulate microbial fermentation and metabolic outputs, leading to the production of SCFAs and tryptophan-derived metabolites. SCFAs, particularly butyrate, contribute to epithelial barrier integrity through multiple mechanisms, including serving as an energy source for colonocytes and upregulating tight junction proteins such as claudin-1, ZO-1 and occludin, thereby maintaining transepithelial resistance and supporting mucin production [10,11]. In parallel, microbial metabolism of tryptophan generates indole and its derivatives including indole-acetic acid (IAA) and indole-lactic acid (ILA), which act as aryl hydrocarbon receptor (AhR) ligands and regulate intestinal barrier integrity through mechanisms such as enhanced mucin sulfation and tight junction modulation, thereby supporting mucosal homeostasis [12,13]. Importantly, these metabolite-mediated pathways converge on the regulation of intestinal permeability. Zonulin, a key modulator of tight junction dynamics, is widely used as a clinical biomarker reflecting changes in barrier integrity [14,15]. Therefore, simultaneous assessment of SCFA production, tryptophan–indole metabolism, and zonulin levels may provide an integrated view of probiotic-mediated modulation of intestinal barrier function [16,17,18,19]. Despite this relevance, integrated clinical evaluations simultaneously assess microbial metabolites and barrier-related markers in response to probiotic interventions remain limited.

In addition to probiotic strains, plant-derived bioactive compounds (phytonutrients) have been reported to influence gut microbial composition and metabolic activity, potentially exerting prebiotic-like effects [20]. While the combination of probiotics with phytonutrients may provide complementary benefits through modulation of microbial ecology, microbiota-derived metabolite production, and intestinal barrier-related pathways, clinical evidence evaluating such formulations remains limited. Furthermore, few intervention studies have simultaneously examined bowel function, microbial metabolites, intestinal barrier-related biomarkers, and gut microbiota composition within a single probiotic–phytonutrient framework, particularly in relation to probiotic complexity and potency [20,21]. To address this gap, we conducted a randomized, double-blind, placebo-controlled trial evaluating probiotic–phytonutrient formulations with different levels of probiotic complexity. The probiotic–phytonutrient blends evaluated in this study were formulated with a combination of *Lacticaseibacillus* and *Bifidobacterium* strains. These specific strains have been shown to maintain microbial homeostasis and promote the production of key metabolites, such as SCFAs and tryptophan-derived indole derivatives, which are crucial for intestinal barrier integrity [22,23,24,25,26,27]. Notably, standardized phytonutrients, including citrus bioflavonoids and dandelion extracts, were incorporated not only to serve as fermentable substrates but also to potentially act synergistically with probiotics to enhance metabolic activity and modulate gut dysbiosis [28,29].

Therefore, the present study aimed to evaluate the effects of two probiotic–phytonutrient blends sharing the same phytonutrient base but differing in microbial complexity—one containing two specific strains and the other a five-strain consortium (including the two strains from the former blend)—on intestinal health in a randomized, double-blind, placebo-controlled trial. We investigated changes in bowel function, fecal SCFA concentrations, the gut barrier-related marker zonulin, tryptophan–indole metabolism, and gut microbiome composition. This study seeks to provide preliminary insights into how probiotic formulation complexity may influence microbiome-associated metabolic responses and intestinal health.

## 2. Materials and Methods

### 2.1. Study Design and Ethics

**Figure 1:**
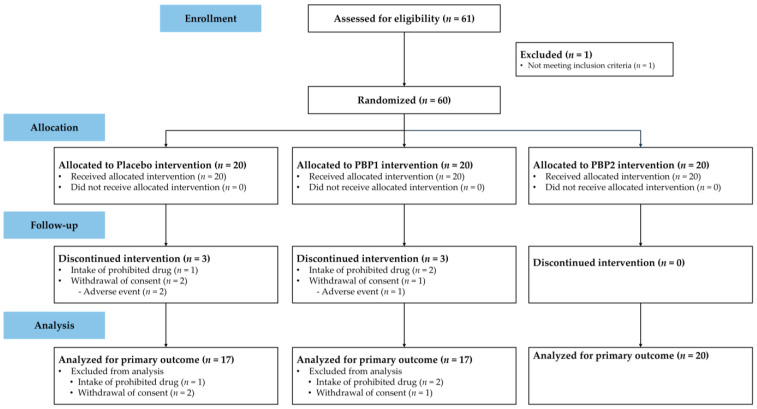
Participant flow chart (CONSORT2025).

This study was a randomized, double-blind, placebo-controlled, parallel-group clinical trial conducted between November 2025 and March 2026 at HEM Pharma. The study was performed in accordance with the principles of the Korea National Institute for Bioethics Policy and was approved by the relevant ethics committee (Approval No. P01-202511-01-048, 24 November 2025). The trial was registered at the Clinical Research Information Service (CRIS; KCT0011874, Registered Date 20 April 2026). Participants were recruited through community advertisements and outpatient clinics and were screened for eligibility based on predefined inclusion and exclusion criteria. A four-week screening period was conducted to confirm stool characteristics, including stool form and frequency, according to the Bristol Stool Form Scale (BSFS). Of the 61 individuals initially assessed for eligibility, 1 was excluded for not meeting the eligibility criteria. The remaining 60 participants were randomly assigned in a 1:1:1 ratio to the placebo (*n* = 20), Probiotic Blend containing Phytonutrients 1 (PBP1, *n* = 20), or Probiotic Blend containing Phytonutrients 2 (PBP2, *n* = 20) groups. Random allocation sequences were generated by an independent statistician using a block randomization method implemented in JMP^®^ v.17.1.0 (SAS Institute Inc., Cary, NC, USA). Investigators did not have access to the randomization sequence until participants were assigned to the intervention groups and statistical analyses were completed. Participants, investigators, outcome assessors, and data analysts were blinded to group assignments throughout the study period. The placebo and intervention products were identical in appearance, packaging, and labeling to maintain blinding. A CONSORT flow diagram (Figure 1) illustrates participant enrollment, allocation, follow-up, and analysis. To ensure compliance and safety, participants recorded daily intake in diary cards, and safety and harms were assessed through adverse event monitoring at each study visit. Adherence was monitored by study personnel through sachet counts and the collection of unused products at the end of the study. Written informed consent was obtained from all participants prior to enrollment. A total of 61 individuals were assessed for eligibility, of whom 1 was excluded for not meeting the inclusion criteria. The remaining 60 participants were randomized equally into the placebo (*n* = 20), PBP1 (*n* = 20), and PBP2 (*n* = 20) groups. During the intervention period, 3 participants in the placebo group and 3 participants in the PBP1 group discontinued the study because of withdrawal of consent or intake of prohibited medications. Consequently, 54 participants completed the study and were included in the primary outcome analysis (placebo, *n* = 17; PBP1, *n* = 17; PBP2, *n* = 20) (Figure 1).

### 2.2. Sample Size Calculation

To determine the minimum number of participants required for this study, the sample size was calculated based on the expected changes in fecal butyrate concentration. The following assumptions were made: a significance level (α) of 0.05 and a statistical power (1 − β) of 80%. Based on a previous clinical trial evaluating metabolic changes following probiotic supplementation, the expected change in butyrate levels after 8 weeks was estimated to be 0.77 ± 0.71 μmol/g for the intervention group and 0.06 ± 0.71 μmol/g for the placebo group [30]. Using G*Power software (Version 3.1.9.7, University of Düsseldorf, Germany) with a 1:1 allocation ratio, the minimum required sample size was determined to be 17 participants per group. To account for a potential dropout rate of approximately 15%, the enrollment target was adjusted to 20 participants per group, resulting in a total of 60 participants for the study.

### 2.3. Supplement Preparation

The capsules administered to the Probiotic Blend containing Phytonutrients 1 (PBP1) contained probiotics, including *Lacticaseibacillus rhamnosus* HEM648 (*Lacticaseibacillus rhamnosus* Nutrilite 648™) and *Lacticaseibacillus paracasei* HEM272 (*Lacticaseibacillus paracasei* Nutrilite 272™), along with phytonutrients composed of dandelion root powder and citrus bioflavonoid complex. Corn starch was used as an excipient, and the formulation was prepared as a hard capsule with a total fill weight of 300 mg. Each capsule was designed to provide a guaranteed viable cell count of 3.0 × 10^9^ CFU. The capsules administered to the Probiotic Blend containing Phytonutrients 2 (PBP2) contained probiotics, including HEM 3 Strains Blend (*Bifidobacterium animalis* subsp. *lactis* IDCC 4301, *Lacticaseibacillus rhamnosus* IDCC 3201, *Lactobacillus acidophilus* IDCC 3302), *Lacticaseibacillus rhamnosus* HEM648 (*Lacticaseibacillus rhamnosus* Nutrilite 648™) and *Lacticaseibacillus paracasei* HEM272 (*Lacticaseibacillus paracasei* Nutrilite 272™), along with the same phytonutrients consisting of dandelion root powder and citrus bioflavonoid complex. Corn starch was used as an excipient, and the formulation was prepared as a hard capsule with a total fill weight of 300 mg. Each capsule was designed to provide a guaranteed viable cell count of 1.0 × 10^10^ CFU. The placebo capsules contained no active ingredients and were composed solely of corn starch, with a total fill weight of 300 mg. The placebo was manufactured as a hard capsule visually indistinguishable from the test products. All capsules were administered once daily, one capsule per dose, taken with water on an empty stomach before meals, and were stored at room temperature.

### 2.4. Study Participants

The participants were men and women over the age 19 years with a body mass index (BMI) ≤ 29.9 kg/m^2^ who were considered generally healthy based on their medical history. The exclusion criteria were as follows: diagnosis of irritable bowel syndrome (IBS); history or presence of chronic diseases, cancer, infection, or surgery that could affect study outcomes; medical or surgical events requiring hospitalization, outpatient treatment, or emergency care within the past year, or any condition deemed unsuitable by the investigator; unstable, acute, or life-threatening conditions; inability to provide stool samples; regular consumption of probiotics, prebiotics, or supplements for energy or mood improvement with unwillingness to discontinue use during the study period and for at least one week prior to screening; adherence to a galactose- or lactose-restricted diet; use of oral antibiotics within the past three months; significant changes in dietary habits within one month prior to screening; intolerance or allergy to sugar alcohols (e.g., mannitol, sorbitol, xylitol, lactulose) or lactose; pregnancy, breastfeeding, or planned pregnancy during the study period; participation in another clinical trial within six months prior to screening; and any other condition that, in the opinion of the investigator, could interfere with safe participation or interpretation of the study results. Sixty participants who met all inclusion and exclusion criteria were enrolled in the study. During the intervention period, participants were instructed to maintain their usual diet and lifestyle, including physical activity, and were prohibited from consuming any additional probiotics, prebiotics, or related functional foods.

### 2.5. Study Procedure

The intervention period lasted 8 weeks, during which participants consumed the assigned product daily. Efficacy endpoints were evaluated at baseline (Week 0), Week 4, and Week 8. Stool patterns were assessed using a 7-day BSFS diary. Gastrointestinal symptoms and bowel habits were evaluated using a gut health questionnaire assessing bowel movement frequency, defecation time, abdominal pain, abdominal bloating, sensation of incomplete evacuation, and postprandial bowel urgency using ordinal or symptom severity scales. Quality of life (QoL) was evaluated using a validated 34-item questionnaire with predefined ordinal or 5-point Likert-type scales [31]. Both total questionnaire scores and individual item responses were analyzed, with higher scores indicating greater symptom severity or QoL impairment. Fecal samples were collected at baseline and Week 8 for the analysis of short-chain fatty acids (SCFAs), zonulin levels, tryptophan metabolites, and gut microbiota composition. Physical measurements and safety assessments, including adverse events, were conducted at each visit.

### 2.6. Fecal Short-Chain Fatty Acids Analysis

All SCFAs were extracted with 0.2 g of fecal sample in 1 mL of dH2O. After vortexing, the mixture was centrifuged at 13,000 rpm for 10 min at 4 °C. The supernatant (150 μL) was collected and transferred to 10 mL of screw cap vial with 150 μL of GC buffer solution, which includes (NH4)2SO4 (Daejung, Siheung, Republic of Korea) and NaH2PO4 (Sigma-Aldrich, St. Louis, MO, USA) with 2-ethylbutric acid (TCI, Tokyo, Japan) as an internal standard [32]. SCFAs were analyzed with headspace sampler-gas chromatography-Flame ionization detector (HSS-GC-FID) which consists of Agilent 7890B GC system equipped with a 7697A headspace sampler and FID (Agilent Technologies, Santa Clara, CA, USA). An HP-innowax capillary column (30 m × 0.32 mm i.d. × 0.50 μL film thickness; Agilent) was used with constant flow of nitrogen as the carrier gas. The operating conditions were as follows: oven temperature, 85 °C; loop temperature 90 °C; transfer lines, 100 °C; FID temperature 250 °C; column temperature was initially at 60 °C, raised to 140 °C at 30 °C/min, then raised to 170 °C at 30 °C, and finally to 180 °C at 40 °C and held for 0.75 min. Data acquisition and operation processing were conducted using ChemStation software (Agilent Technologies). SCFAs were identified and quantified using standard compounds.

### 2.7. Fecal Zonulin Measurement

Fecal samples were collected using the IDK^®^ Stool Collection Kit (K6998SAS; Immundiagnostik AG, Bensheim, Germany) and stored according to the manufacturer’s instructions until analysis. Fecal zonulin concentrations were measured using a commercially available enzyme-linked immunosorbent assay (ELISA) kit (IDK^®^ Zonulin ELISA, KR5600; Immundiagnostik AG, Bensheim, Germany) according to the manufacturer’s protocol. Briefly, fecal samples were extracted and diluted using the buffer system provided with the kit. Prepared samples, standards, and controls were added to microplate wells coated with anti-zonulin antibodies and incubated according to the assay protocol. After washing, an enzyme conjugate was added, followed by substrate solution for color development. The reaction was stopped, and absorbance was measured at 405 nm with a reference wavelength of 620 nm using a Multiskan™ GO microplate spectrophotometer (Thermo Fisher Scientific Oy, Vantaa, Finland). A standard curve was generated using a four-parameter logistic (4PL) regression model implemented in R (version 4.3.0; R Foundation for Statistical Computing, Vienna, Austria) with the drc package (version 3.0-1). Sample concentrations were interpolated from the fitted curve within the standard range, and values below the limit of quantification (LOQ) were assigned the LOQ value for subsequent analysis. Zonulin concentrations were expressed as ng/mL.

### 2.8. Tryptophan Metabolism-Related Metabolites Analysis

Stool samples (50 mg) were extracted with 50% methanol (Fisher Scientific, Hampton, NH, USA) (1 mL) in a 2 mL microcentrifuge tube. The samples were vortexed for 10 min and sonicated for 10 min at room temperature. After centrifugation at 13,000 rpm for 10 min at 4 °C, the supernatant was filtered through a 0.2 μm polytetrafluoroethylene syringe filter (Alwsci® Technologies, Hangzhou, China). The filtered supernatant was used for tryptophan metabolite analysis.

Tryptophan metabolism-related metabolites quantitative analysis was carried out using a Shimadzu LCMS-8050 triple quadrupole tandem mass spectrometry detector (Shimadzu, Kyoto, Japan) and an ACQUITY UPLC HSS T3 column (150 × 2.1 mm I.D., 1.8 µm particle size; Waters, Torrance, CA, USA). The column temperature was set at 40 °C. The mobile phases A and B consisted of 0.1% formic acid (Supelco, Bellefonte, PA, USA) (*v*/*v*) in water and 0.1% formic acid (*v*/*v*) in acetonitrile (Daejung, Siheung, Republic of Korea), respectively. Gradient elution was programmed as follows: 5% of mobile phase B for 0.0–1.0 min; 5–100% of B for 1.0–11.0 min; 100% of B for 11.0–12.0 min; 100–5% of B for 12.0–13.0 min; and 5% of B for 13.0–14.0 min for re-equilibration. The flow rate was 0.3 mL/min, and the injection volume was 1 μL. All analytes were ionized by electrospray ionization, primarily in the positive ion mode, with negative ion mode used only for norepinephrine, and detected in multiple reaction monitoring (MRM) mode. A total of 36 metabolites related to neurotransmitters, tryptophan metabolism, indole derivatives, and kynurenine pathway metabolites were included in the analytical panel. The polarity and quantifier MRM transition for each target metabolite are provided in Supplementary Table S1. Among the targeted metabolites, compounds that were reliably detected and quantified in the fecal samples were used for subsequent statistical analysis.

### 2.9. Gut Microbiota Analysis

Genomic DNA was extracted from fecal samples using the Mag-Bind^®^ Universal Pathogen Kit (Omega Bio-tek, Norcross, GA, USA) according to the manufacturer’s instructions. Briefly, fecal pellets were resuspended in 275 μL of SLX-Mlus buffer and subjected to mechanical lysis using a MixerMill MM400 bead-beating system (Retsch, Haan, Germany). DNA was subsequently purified and eluted. The V3–V4 hypervariable regions of the bacterial 16S rRNA gene were amplified using the following primers: forward primer (5′-TCGTCGGCAGCGTCAGATGTGTATAAGAGACAGCCTACGGGNGGCWGCAG-3′) and reverse primer (5′-GTCTCGTGGGCTCGGAGATGTGTATAAGAGACAGGACTACHVGGGTATCTAATCC-3′). PCR amplification was performed using 2× KAPA HiFi HotStart ReadyMix (Roche, Basel, Switzerland) with the following cycling conditions: initial denaturation at 95 °C for 3 min; 25 cycles of 95 °C for 30 s, 55 °C for 30 s, and 72 °C for 30 s; followed by a final extension at 72 °C for 5 min. PCR amplicons were purified using HiAccuBeads (AccuGene, Incheon, Republic of Korea) and a magnetic stand. Indexing PCR was subsequently carried out using IDT indexing primers (Integrated DNA Technologies, Coralville, IA, USA), 2× KAPA HiFi HotStart ReadyMix, and PCR-grade water. Cycling conditions consisted of an initial denaturation at 95 °C for 3 min, followed by 8 cycles of 95 °C for 30 s, 55 °C for 30 s, and 72 °C for 30 s, with a final extension at 72 °C for 5 min and a hold at 4 °C. Following purification, library concentrations were measured using a Qubit 4.0 fluorometer (Thermo Fisher Scientific, Waltham, MA, USA) with the dsDNA High Sensitivity Assay Kit. Sequencing was performed on the Illumina MiSeq platform (Illumina, San Diego, CA, USA) to generate paired-end reads targeting the V3–V4 region. Raw sequencing data were demultiplexed based on unique sample-specific barcodes. Sequence data were processed using QIIME2 (version 2021.2) following standard analytical workflows. Primer trimming, quality filtering, denoising, paired-end merging, and chimera removal were conducted using the DADA2 plugin (qiime dada2 denoise-paired), generating amplicon sequence variants (ASVs). Forward and reverse reads were trimmed by 17 bp and 21 bp, respectively, to remove primer sequences. Based on quality score profiles, reads were truncated at 270 bp (forward) and 220 bp (reverse) to retain high-quality sequences while minimizing sequencing errors. All other parameters were set to default values. Taxonomic classification of ASVs was performed using a naïve Bayes classifier trained on the SILVA reference database (version 138), trimmed to the V3–V4 region amplified in this study. Classification was conducted using the qiime feature-classifier classify-sklearn plugin with default confidence thresholds.

After DADA2 processing, all subsequent microbiome analyses were carried out in R. Alpha-diversity indices, including Chao1, Observed ASVs, Shannon, and Simpson, were calculated using the phyloseq package (version 1.46.0), and changes from baseline were calculated as Δ values (post-intervention minus baseline) for downstream comparisons and visualization. For beta-diversity analysis, the feature table was rarefied to the lowest sequencing depth across samples. Microbial community differences were evaluated using multiple distance metrics, including weighted UniFrac, unweighted UniFrac, Bray–Curtis, and Jaccard distances, followed by visualization with principal coordinates analysis (PCoA). Differential abundance testing was conducted using DESeq2 (version 1.42.1), and relative abundance changes from baseline were examined for selected taxa.

### 2.10. Statistical Analysis

All statistical analyses were performed using JMP^®^ v.17.1.0 (SAS Institute Inc., Cary, NC, USA). Continuous variables are presented as mean ± standard deviation (SD) or standard error (SE), as appropriate, while categorical variables are expressed as frequencies and percentages. Normality of continuous variables was assessed using the Shapiro–Wilk test. For within-group comparisons, paired *t*-tests were used for normally distributed data, and the Wilcoxon signed-rank test was applied for non-normally distributed data. For between-group comparisons, independent *t*-tests or one-way analysis of variance (ANOVA) were used for normally distributed data, while the Mann–Whitney U test (Wilcoxon rank-sum) or Kruskal–Wallis test was used for non-normally distributed data. Categorical variables, including stool type distributions, were analyzed using the chi-square test or Fisher’s exact test, as appropriate. For within-group comparisons of paired categorical variables over time (e.g., baseline vs. Week 4 or Week 8), McNemar’s test was used. Changes from baseline were calculated as the difference between post-intervention and baseline values. When multiple comparisons were performed, post hoc analyses were conducted using Tukey’s test or Dunn’s test with Bonferroni correction, as appropriate. Subgroup analyses were performed according to baseline body mass index (BMI) categories (<25.0 kg/m^2^ and 25.0 ≤ BMI < 30.0 kg/m^2^). Correlation analyses were performed using linear regression, and model significance was evaluated using the F-test. All statistical tests were two-sided, and a *p*-value < 0.05 was considered statistically significant. For microbiome data, changes from baseline in alpha-diversity metrics (Chao1, Observed ASVs, Shannon, and Simpson indices) and relative abundance were compared between groups using the Wilcoxon rank-sum test in R. Beta-diversity metrics (weighted UniFrac, unweighted UniFrac, Bray–Curtis, and Jaccard distances) were analyzed using permutational multivariate analysis of variance (PERMANOVA) in the vegan R package (version 2.6.8), and Group × Time interaction effects were evaluated. Differential abundance analysis was performed using DESeq2 with raw (non-rarefied) ASV count tables incorporating group-by-time interaction effects. The log2 fold changes represent the Group × Time interaction coefficients, reflecting differential changes from baseline to Week 8 in PBP1 and PBP2 relative to placebo. Measurements below the limit of detection were considered missing and were not included in statistical analyses.

## 3. Results

### 3.1. Participant Characteristics

A total of 54 participants were included in the final efficacy analysis and assigned to the PBP1 group (*n* = 17), PBP2 group (*n* = 20), or placebo group (*n* = 17). Baseline demographic and clinical characteristics are summarized in Table 1. There were no statistically significant differences among the groups in terms of age, sex distribution, body mass index (BMI), or stool consistency at baseline (all *p* > 0.05). The mean age was comparable across groups (PBP1: 40.18 ± 10.34 years; PBP2: 41.10 ± 9.53 years; placebo: 39.47 ± 8.80 years). The proportion of female participants ranged from 47.06% to 60.00% across groups. Baseline BMI was also similar among the groups (PBP1: 24.23 ± 4.02 kg/m^2^; PBP2: 23.36 ± 3.04 kg/m^2^; placebo: 22.85 ± 3.18 kg/m^2^), with no significant differences in BMI category distribution (<25.0 vs. 25.0–30.0 kg/m^2^). Stool consistency scores and the distribution of constipation, normal stool, and diarrhea categories were comparable across groups. Baseline fecal biomarker levels, including short-chain fatty acids (SCFAs), butyrate, and zonulin, did not differ significantly between groups. Mean SCFA levels were 67.94 ± 20.94 μg/g in the PBP1 group, 68.01 ± 18.42 μg/g in the PBP2 group, and 82.33 ± 26.95 μg/g in the placebo group. Similarly, butyrate and zonulin levels were not significantly different among the groups. Overall, these findings indicate that the treatment groups were well balanced at baseline, allowing for appropriate comparison of post-intervention outcomes.

### 3.2. Assessments of Gut Health and Quality of Life

#### 3.2.1. Changes in Stool Patterns

Based on the 7-day Bristol Stool Form Scale (BSFS) diary (Table 2), both PBP1 and PBP2 groups showed increases in the proportion of normal stool types (BSFS types 3–5) over the intervention period. In the PBP1 group, the proportion of normal stool types increased from 74.10 ± 22.80% at baseline to 88.07 ± 17.45% at Week 8, with a significant within-group change (*p* = 0.0005). Similarly, the PBP2 group showed a significant increase in normal stool types from 67.44 ± 29.06% at baseline to 79.67 ± 20.74% at Week 8 (*p* = 0.0304). In contrast, no significant changes were observed in the placebo group over time.

**Figure 2:**
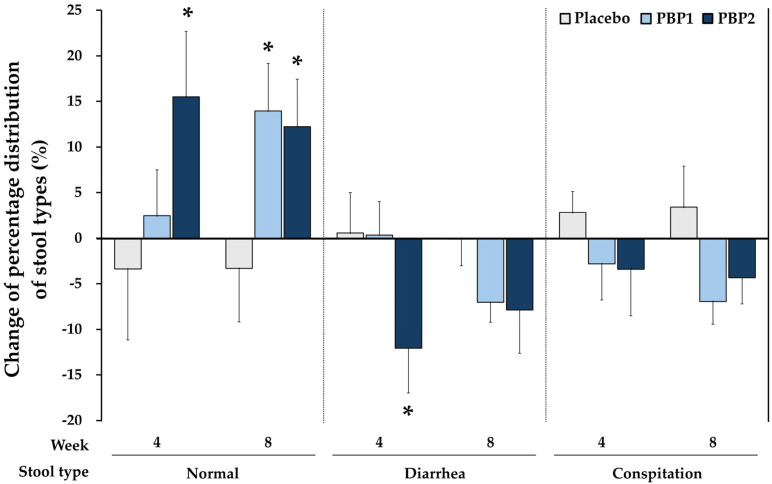
Effects of PBP1 and PBP2 supplementation on stool type distribution assessed by the 7-day Bristol Stool Form Scale (BSFS). Changes in the proportion of stool types from baseline to Weeks 4 and 8 are shown for the placebo, PBP1, and PBP2 groups. Bars represent mean ± standard error (SE). Both PBP1 and PBP2 groups demonstrated a significant increase in the proportion of normal stool types (BSFS types 3–5) compared with the placebo group at Week 8 (PBP1: +13.96%, *p* = 0.0151; PBP2: +12.22%, *p* = 0.0226). The PBP2 group also showed a significant increase at Week 4 (+15.50%, *p* = 0.0360). Additionally, the PBP2 group exhibited a significant reduction in diarrheal stool types (BSFS types 6–7) at Week 4 compared with placebo (*p* = 0.0468), with a sustained decreasing trend at Week 8. * *p* < 0.05 vs. placebo.

As shown in Figure 2, both PBP1 and PBP2 groups demonstrated significant improvements in stool type distribution compared with the placebo group. At Week 8, the PBP1 group showed a significant increase in the proportion of normal stool types compared with the placebo group (+13.96%, *p* = 0.0151). The PBP2 group also exhibited significant increases in normal stool types at both Week 4 (+15.50%, *p* = 0.0360) and Week 8 (+12.22%, *p* = 0.0226) compared with the placebo group. In addition, the PBP2 group showed a lower proportion of diarrheal stool types (BSFS types 6–7) at Week 4 compared with the placebo group (*p* = 0.0468), with a similar pattern observed at Week 8.

#### 3.2.2. Changes in Gastrointestinal Symptoms and Bowel Habits

Changes in gastrointestinal symptoms and bowel habits are summarized in Table 3. Bowel movement frequency did not significantly differ between the intervention groups and placebo at Week 8. However, the PBP2 group showed a significantly lower bowel movement frequency score than the placebo group at Week 4 (*p* = 0.0015). Defecation time, abdominal pain, abdominal bloating, and sensation of incomplete evacuation did not significantly differ between groups during the intervention period. Notably, urgency of postprandial bowel movements was significantly improved in the PBP1 group compared with the placebo group at both Week 4 and Week 8 (*p* = 0.0066 and *p* = 0.0029, respectively). A decreasing trend was also observed in the PBP2 group, although statistical significance was not reached.

#### 3.2.3. Changes in Quality of Life

Quality of life (QoL) scores were comparable among the three groups at baseline (placebo: 45.00 ± 18.34; PBP1: 44.82 ± 14.29; PBP2: 44.40 ± 17.72; *p* > 0.05). During the 8-week intervention period, no statistically significant differences in total QoL score changes were observed between groups at either Week 4 or Week 8 (all *p* > 0.05). Exploratory analyses of individual questionnaire items showed numerical improvements in several bowel discomfort- and social burden-related items in the intervention groups compared with placebo, although these findings were limited to selected questionnaire items.

### 3.3. Changes in Fecal SCFAs Levels

**Figure 3:**
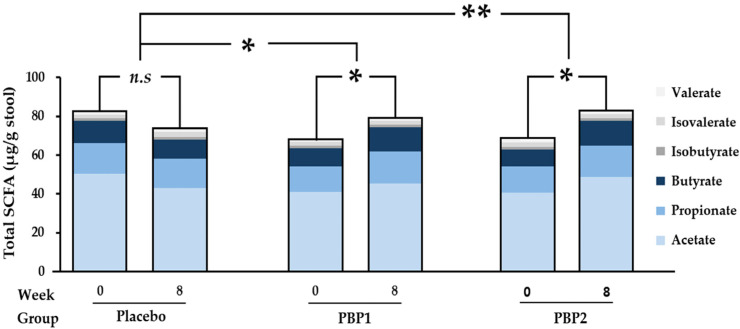
Effects of PBP1 and PBP2 supplementation on total fecal short-chain fatty acid (SCFA) concentrations and composition. Total SCFAs levels and relative composition (acetate, propionate, and butyrate) at baseline (Week 0) and Week 8 are shown for each group. Bars represent mean values, and stacked segments indicate individual SCFAs components. No significant changes were observed in the placebo group over time. In contrast, both PBP1 and PBP2 groups exhibited significant increases in total SCFAs concentrations from baseline to Week 8. At Week 8, total SCFAs levels were significantly higher in both intervention groups compared with the placebo group (*p* < 0.05). The increase was consistently observed across major SCFAs components, including butyrate and acetate. * *p* < 0.05, ** *p* < 0.01 vs. placebo.; n.s., not significant.

**Figure 4:**
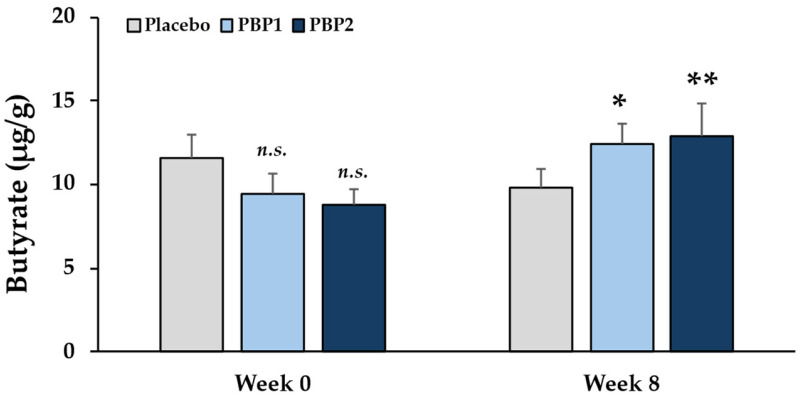
Changes in fecal butyrate levels following PBP1 and PBP2 supplementation. Fecal butyrate concentrations at baseline (Week 0) and Week 8 are shown for the placebo, PBP1, and PBP2 groups. Bars represent mean ± standard error (SE). At Week 8, both PBP1 and PBP2 groups demonstrated significant increases in butyrate levels compared with the placebo group, whereas no significant differences were observed at baseline. Although absolute values are displayed in the figure, statistical comparisons were performed using the change values. * *p* < 0.05, ** *p* < 0.01 vs. placebo.; n.s., not significant.

Fecal SCFAs analysis showed that total SCFAs concentrations significantly increased from baseline to Week 8 in both the PBP1 and PBP2 groups, whereas no significant change was observed in the placebo group (Figure 3, Supplementary Figure S1). At Week 8, total SCFAs levels were significantly higher in the PBP1 and PBP2 groups compared with the placebo group (*p* < 0.05). From baseline, total SCFAs levels increased by 11.19 ± 18.29 μg/g in the PBP1 group (*p* = 0.0285) and by 14.90 ± 29.14 μg/g in the PBP2 group (*p* = 0.0062), corresponding to increases of 20.01% and 24.65%, respectively. In contrast, the placebo group showed a decrease of 4.82%. In addition to the overall increase, butyrate and acetate levels were significantly increased in the intervention groups compared with the placebo group (Supplementary Figure S2). Butyrate levels increased from 9.47 ± 1.19 to 12.42 ± 1.20 μg/g in the PBP1 group and from 8.82 ± 0.91 to 12.86 ± 1.97 μg/g in the PBP2 group, whereas the placebo group showed a decrease from 11.58 ± 1.40 to 9.80 ± 1.11 μg/g (Figure 4; *p* < 0.05).

### 3.4. Changes in Fecal Zonulin

**Figure 5:**
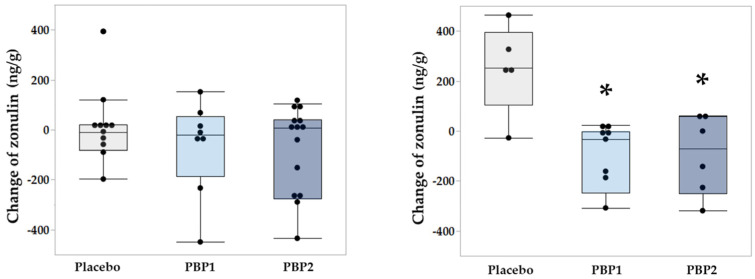
Changes in fecal zonulin levels according to baseline BMI subgroups. Participants were stratified into BMI < 25.0 kg/m^2^ and 25.0 ≤ BMI < 30.0 kg/m^2^ groups. Box plots display median and interquartile range, with individual data points overlaid. In participants with BMI < 25.0 kg/m^2^, both PBP1 and PBP2 groups showed a decreasing trend in fecal zonulin levels; however, no statistically significant differences were observed compared with placebo. In contrast, in participants with 25.0 ≤ BMI < 30.0 kg/m^2^, significant reductions in fecal zonulin levels were observed in both the PBP1 and PBP2 groups compared with the placebo group. * *p* < 0.05 vs. placebo.

Fecal zonulin levels did not differ significantly among the groups at baseline. After the intervention, both the PBP1 and PBP2 groups showed a decreasing trend in zonulin levels; however, these changes were not statistically significant compared with the placebo group (Supplementary Figure S3). In subgroup analyses, participants with BMI < 25.0 kg/m^2^ showed a similar decreasing trend without statistical significance. In contrast, in participants with 25.0 ≤ BMI < 30.0 kg/m^2^, significant reductions in zonulin levels were observed in both the PBP1 (−155.62 ± 228.95 ng/g) and PBP2 (−95.55 ± 158.64 ng/g) groups compared with the placebo group (*p* < 0.05) (Figure 5).

### 3.5. Changes in Fecal Tryptophan and Indole Metabolites

**Figure 6:**
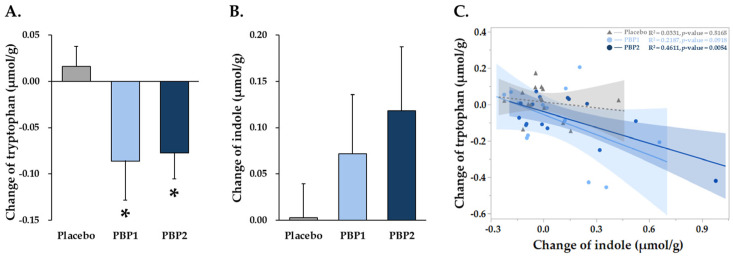
Modulation of tryptophan metabolism toward indole production following PBP1 and PBP2 supplementation. (**A**,**B**) Changes (Δ, Week 8–Baseline) in fecal tryptophan (**A**) and indole (**B**) levels across groups. Bars represent mean ± standard error (SE). Tryptophan levels were significantly reduced in both PBP1 and PBP2 groups compared with placebo (*p* = 0.0285 and *p* = 0.0456, respectively), while indole levels showed an increasing trend in the intervention groups. (**C**): Correlation analysis between changes in tryptophan and indole levels. An inverse relationship was observed, indicating that increased indole production is associated with decreased tryptophan levels. This relationship showed a trend toward an inverse association in the PBP1 group and a statistically significant inverse correlation in the PBP2 group (R^2^ = 0.4611, *p* = 0.0054), indicating a stronger inverse association in the PBP2 group. Linear regression lines with 95% confidence intervals are shown. Participants with measurements below the limit of detection were excluded from the analyses. For tryptophan analysis, 3 participants were excluded (placebo, *n* = 1; PBP2, *n* = 2). For indole analysis, 7 participants were excluded (placebo, *n* = 1; PBP1, *n* = 3; PBP2, *n* = 3). * *p* < 0.05 vs. placebo.

Tryptophan and indole levels did not differ significantly among the groups at baseline. Following intervention, fecal tryptophan levels were significantly reduced in both PBP groups compared with the placebo group. The mean change in tryptophan was −0.0862 ± 0.1743 μmol/g in the PBP1 group and −0.0771 ± 0.1201 μmol/g in the PBP2 group, both showing statistically significant decreases relative to placebo (*p* = 0.0285 and *p* = 0.0456, respectively) (Figure 6A). In contrast, indole metabolites, including indole, indole–acetic acid (IAA), indole-lactic acid (ILA), and indolepropionic acid (IPA), showed an overall increasing trend following PBP1 and PBP2 intake. For indole, the mean change was 0.0719 ± 0.2398 μmol/g in the PBP1 group and 0.1184 ± 0.2842 μmol/g in the PBP2 group, compared with 0.0027 ± 0.1468 μmol/g in the placebo group, indicating a clear increasing tendency (Figure 6B). Similarly, IAA, ILA, and IPA levels also exhibited consistent upward trends in both PBP1 and PBP2 groups relative to placebo (Supplementary Figure S4). Correlation analysis between tryptophan and indole levels demonstrated an inverse relationship, whereby increased fecal indole levels were associated with decreased tryptophan levels. This relationship showed a trend toward an inverse association in the PBP1 group (R^2^ = 0.2187, *p* = 0.0918), whereas a significant correlation was observed in the PBP2 group (R^2^ = 0.4611, *p* = 0.0054) (Figure 6C).

### 3.6. Changes in Gut Microbiota

**Figure 7:**
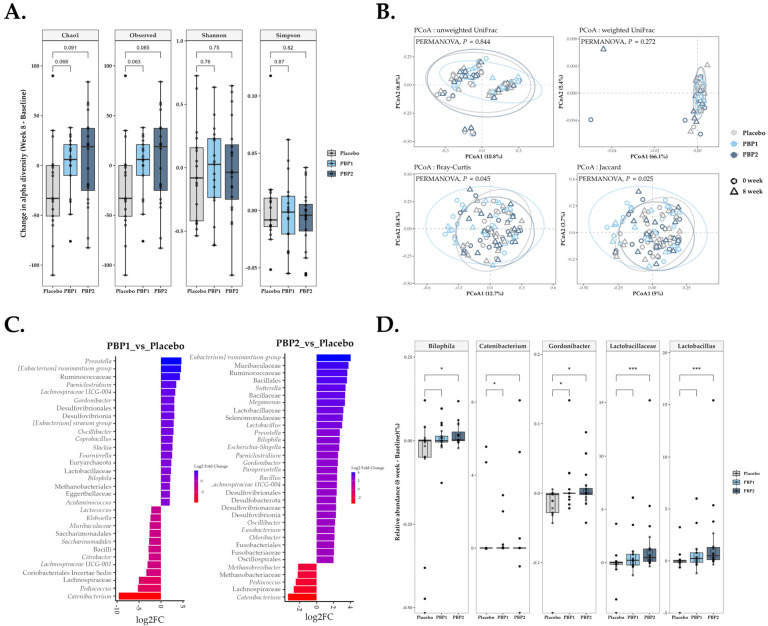
Effects of PBP1 and PBP2 supplementation on gut microbiome diversity and composition. (**A**) Changes in alpha diversity indices (Chao1, observed, Shannon, and Simpson) from baseline to Week 8 across placebo, PBP1, and PBP2 groups. Alpha diversity indices were calculated at the ASV level. Statistical comparisons were performed using the Wilcoxon rank-sum test. No significant differences were observed, indicating preservation of overall microbial diversity. (**B**) Principal coordinate analysis (PCoA) based on unweighted UniFrac, weighted UniFrac, Bray–Curtis, and Jaccard distances metrics. No significant differences were observed in phylogenetic structure (UniFrac), whereas significant differences in Bray–Curtis and Jaccard distances suggest compositional shifts in microbial communities over time between groups. The corresponding *p*-values were derived from the Group × Time interaction term in the PERMANOVA model. (**C**) Differential abundance analysis showing log2 fold changes representing Group × Time interaction coefficients from DESeq2, reflecting differential changes from baseline to Week 8 in PBP1 and PBP2 relative to placebo. Selective enrichment of specific taxa was observed, indicating targeted microbiome modulation. (**D**) Relative abundance changes in key taxa, including *Bilophila*, *Catenibacterium*, *Lactobacillus*, Lactobacillaceae, and *Gordonibacter*. Significant increases in probiotic-associated taxa confirm successful microbial engraftment. Overall, these findings indicate that probiotic supplementation preserves global microbial structure while inducing selective compositional changes, potentially associated with functional metabolic shifts. Statistical comparisons were performed using the Wilcoxon rank-sum test. * *p* < 0.05, *** *p* < 0.001 vs. placebo.

To evaluate changes in gut microbiota, fecal samples were analyzed using 16S rRNA gene sequencing. We first assessed overall microbial diversity. Alpha diversity analysis showed no significant differences in Chao1, observed, Shannon, and Simpson indices among the placebo, PBP1, and PBP2 groups (Figure 7A). Chao1 and observed richness analyses showed minimal changes at the phylum and class levels, while progressive differences emerged at the order, family, and genus levels (Supplementary Figure S5). These genus-level changes are likely associated with the administered probiotic formulations (PBP1 and PBP2). Beta diversity analysis revealed no significant differences in phylogenetic structure based on unweighted and weighted UniFrac distances (Figure 7B). In contrast, Bray–Curtis and Jaccard analyses showed significant Group × Time interaction effects in microbial community composition, suggesting non-phylogenetic compositional shifts following intervention. Differential abundance analysis demonstrated selective changes in specific microbial taxa following intervention (Figure 7C). Notably, several taxa associated with carbohydrate fermentation and microbial metabolism were increased, while others showed reduced abundance, indicating targeted microbiome modulation. Compared to the placebo group, both PBP1 and PBP2 groups exhibited increased abundance of probiotic-associated taxa, including Lactobacillaceae, as well as other taxa such as *Eubacterium ruminantium* group, *Prevotella*, *Gordonibacter*, and *Oscillibacter*. In the PBP2 group, additional changes were observed in taxa including Muribaculaceae, Bacillales, and Bacillaceae, suggesting a broader compositional shift compared to PBP1. In contrast, *Catenibacterium* and *Pediococcus* showed a decreasing trend compared to the placebo group. To further illustrate representative taxa showing significant between-group differences, changes in *Bilophila*, *Catenibacterium*, *Gordonibacter*, Lactobacillaceae, and *Lactobacillus* are presented in Figure 7D. Compared with the placebo group, the PBP2 group exhibited significantly greater increases in *Gordonibacter*, Lactobacillaceae, and *Lactobacillus* abundance, while significant differences in *Bilophila* and *Catenibacterium* were also observed between groups. These findings indicate that probiotic–phytonutrient supplementation induced selective compositional changes in specific microbial taxa despite the preservation of overall microbial diversity.

### 3.7. Changes in Human Intestinal Organoid-Derived Monolayer Model

To further investigate potential mechanisms underlying the clinical findings, exploratory in vitro experiments were conducted using PBP2, which showed relatively more consistent and statistically significant changes across several biomarker outcomes in this clinical study compared with PBP1. A human intestinal organoid-derived monolayer model exhibiting robust barrier function and epithelial differentiation was established, as confirmed by TEER measurements and epithelial markers (Supplementary Figure S6). In this model, PBP2-derived conditioned media increased TEER, reduced paracellular permeability, and protected against cytokine (IFN-γ and TNF-α)-induced barrier disruption. In addition, PBP2 increased the expression of tight junction-associated proteins, including ZO-1, ZO-3, and CLDN8, while reducing the expression of the pore-forming protein claudin-2 (Supplementary Figures S7 and S8). These findings are consistent with previous reports linking ZO-1 and ZO-3 to enhanced barrier function and claudin-2 to increased permeability [33,34,35,36] and suggest that microbiota-derived metabolites may contribute to reinforcement of epithelial barrier integrity.

## 4. Discussion

In this randomized, double-blind, placebo-controlled trial, supplementation with two probiotic–phytonutrient blends (PBP1 and PBP2) improved bowel function, as reflected by an increased proportion of normal stool types, and was associated with coordinated changes in microbiome-derived metabolites. Specifically, increases in fecal short-chain fatty acids (SCFAs), including butyrate and acetate, together with shifts in tryptophan metabolism, were observed. Notably, an inverse relationship between tryptophan and indole was observed, reaching statistical significance in the PBP2 group and showing a similar trend in the PBP1 group, suggesting enhanced microbial metabolic activity. These metabolic changes were accompanied by reductions in the gut barrier-related marker zonulin in a BMI-dependent manner. Microbiome analysis indicated modest changes in bacterial diversity, while PBP1 and PBP2 intervention was associated with increased abundances of *Lactobacillus*, Lactobacillaceae, and *Gordonibacter* compared to the placebo group. Collectively, these findings suggest a potential role of microbiome-mediated metabolic modulation in intestinal health. No severe adverse events were observed during the study (Supplementary Table S2).

One of the key findings of the present study was the improvement in stool patterns, characterized by an increased proportion of normal stool forms (Bristol Stool Form Scale types 3–5). The proportion of normal stools increased significantly in both the PBP1 and PBP2 groups following supplementation (13.96%, *p* = 0.0151 and 12.22%, *p* = 0.0226, respectively), whereas no meaningful change was observed in the placebo group (−3.32%, *p* = 0.5321). These results suggest that PBP1 and PBP2 supplementation may contribute to the normalization of bowel habits by increasing normal stool types while reducing abnormal stool forms. In addition to changes in stool patterns, some gastrointestinal symptom-related questionnaire items showed improvement following supplementation. Notably, urgency of postprandial bowel movements was significantly reduced in the PBP1 group compared with the placebo group. These findings are consistent with previous evidence indicating that alterations in gut microbiota composition are closely associated with bowel function. Patients with irritable bowel syndrome (IBS) have been reported to exhibit reduced abundances of *Bifidobacterium* and *Lactobacillus* species [37], and supplementation with probiotic strains belonging to these genera has been shown to alleviate IBS symptom severity [38,39]. Prior studies have demonstrated that both single-strain and multi-strain probiotic interventions can improve bowel habits, stool consistency, and transit time. For example, supplementation with *Lacticaseibacillus rhamnosus* [24], *Lactobacillus acidophilus* [40], and *Lacticaseibacillus paracasei* [41] have been associated with improvements in defecation patterns, while multi-strain formulations have shown similar benefits [42]. Mechanistically, these probiotic strains may contribute to gut function through mucosal adhesion, colonization, and modulation of the intestinal environment, which could underlie their beneficial effects on bowel habits and gastrointestinal health [43,44]. Clinical studies further support that *L. paracasei* supplementation may improve stool consistency in individuals with constipation or diarrhea, including patients with IBS [14,41]. In contrast, overall quality of life scores did not significantly differ between groups during the intervention period, although several individual questionnaire items related to bowel discomfort and social burden showed significant improvements in the intervention groups. Taken together, the observed improvements in stool form and selected gastrointestinal symptoms in the present study are well aligned with existing literature, supporting the role of probiotic supplementation in modulating gut function and promoting healthy bowel patterns.

These clinical improvements were accompanied by an increase in total fecal SCFAs, including acetate and butyrate, which are known to be associated with intestinal motility and epithelial homeostasis. Among SCFAs, butyrate is a key microbial metabolite produced through fermentation and serves as a primary energy source for colonocytes. In addition, butyrate has been reported to play an important role in regulating intestinal motility, modulating water and electrolyte absorption, and maintaining mucosal function, all of which are closely linked to the formation of normal stool consistency [45,46]. Previous metagenomic and functional studies have suggested that probiotic supplementation enhances the production of SCFAs such as butyrate, acetate, and propionate, strengthens epithelial barrier integrity, and exerts anti-inflammatory effects through modulation of cytokine secretion and immune cell activity [16,45]. Furthermore, increased fecal butyrate levels have been reported following multi-strain probiotic supplementation [47], consistent with the findings of the present study. However, participants were instructed to maintain their habitual diet and lifestyle throughout the study period, and detailed dietary intake was not systematically monitored or controlled. Because dietary composition can substantially influence SCFA production and tryptophan metabolism, dietary variation may have contributed to inter-individual variability in metabolite profiles. Nevertheless, given the randomized, placebo-controlled design of the study, such variability would be expected to be relatively balanced across the intervention groups. Therefore, the observed increase in butyrate may contribute to the normalization of bowel habits by improving epithelial function and the luminal environment. Given its well-established role in maintaining epithelial barrier integrity [33], the increase in butyrate may also suggest a potential link between microbial metabolic activity and intestinal barrier function. Building on these findings, we further investigated whether changes in microbial metabolism may be associated with intestinal barrier regulation by examining fecal tryptophan–indole metabolism and zonulin levels.

In the present study, multi-strain probiotic supplementation was associated with a shift in tryptophan metabolism, characterized by decreased fecal tryptophan levels and a concomitant increase in indole metabolites. Notably, a significant inverse correlation between tryptophan and indole levels was observed in the PBP2 group, suggesting enhanced microbial conversion of tryptophan into indole derivatives. Indole and its derivatives are well-recognized microbial signaling molecules that contribute to intestinal homeostasis. These metabolites have been shown to activate host pathways such as the pregnane X receptor (PXR) and aryl hydrocarbon receptor (AhR), leading to enhanced expression of genes involved in mucosal barrier integrity, including tight junction proteins and mucin production [48,49,50]. In this context, the observed increase in indole metabolites following PBP1 and PBP2 supplementation may reflect a functionally relevant metabolic shift toward barrier-supportive signaling, consistent with the concurrent reduction in zonulin levels.

Fecal zonulin levels showed a decreasing trend following PBP1 and PBP2 supplementation, with a statistically significant reduction observed particularly among participants with BMI ≥ 25.0 kg/m^2^. Zonulin is a physiological modulator that regulates the reversible opening of intestinal epithelial tight junctions, and its activation has been shown to induce cytoskeletal rearrangement and redistribution of tight junction proteins, leading to increased intestinal permeability [15,51,52]. These changes are closely associated with the so-called “leaky gut” condition and chronic inflammatory states [14]. Therefore, the reduction in zonulin observed in the present study may be interpreted as indicative of supportive modulation of the intestinal epithelial barrier homeostasis and decreased intestinal permeability. These findings are consistent with previous clinical and meta-analytic studies. For example, combined supplementation with probiotics and vitamin D has been shown to significantly reduce serum zonulin levels and improve intestinal barrier function in patients with irritable bowel syndrome [42], while meta-analytic evidence indicates that probiotic or synbiotic interventions may improve intestinal permeability through reductions in zonulin levels [53]. In addition, a randomized controlled trial in overweight and obese adults reported decreased zonulin levels alongside improvements in body fat mass and trends toward increased SCFA production following probiotic supplementation [54]. These observations are in line with the present study, in which a more pronounced reduction in zonulin and a concomitant increase in butyrate were observed in participants with BMI ≥ 25.0 kg/m^2^. Higher BMI has been associated with altered gut microbiota composition, increased intestinal permeability, and insulin resistance [55], suggesting that individuals with this metabolic background may exhibit greater responsiveness to probiotic interventions. Accordingly, the BMI-dependent reduction in zonulin observed in this study may reflect an amplified response in individuals with relatively compromised baseline barrier function. Accordingly, the BMI-dependent reduction in zonulin observed in this study may reflect an amplified response in individuals with relatively compromised baseline barrier function. However, as this observation is based on subgroup analysis, it should be interpreted with caution and requires further validation. In the present study, zonulin was used as a surrogate marker associated with intestinal barrier function; however, additional barrier-related biomarkers were not evaluated. Furthermore, although the observed associations between zonulin changes and microbiota-derived metabolites, including tryptophan and indole, suggest a potential link between microbial metabolic activity and intestinal barrier-related markers, these findings should be interpreted cautiously given the exploratory nature of the analyses. Although fecal zonulin is widely used as a surrogate marker of intestinal permeability, its analytical specificity and interpretation remain under discussion.

**Figure 8:**
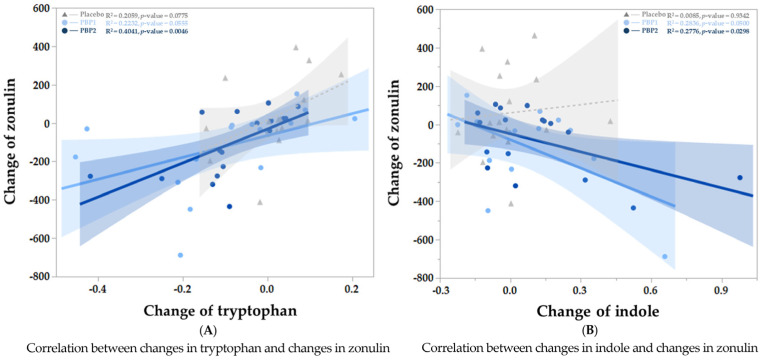
Associations between changes in tryptophan, indole, and zonulin following intervention. Decreased zonulin levels were associated with reduced tryptophan and increased indole levels, consistent with enhanced microbial conversion of tryptophan into indole metabolites. (**A**) Correlation between changes in tryptophan and changes in zonulin. A significant positive association was observed in the PBP2 group (R^2^ = 0.4041, *p* = 0.0046). PBP1 showed a positive trend that did not reach statistical significance (R^2^ = 0.2232, *p* = 0.0555), while the placebo group showed no significant association. (**B**) Correlation between changes in indole and changes in zonulin. A significant inverse association was observed in the PBP2 group (R^2^ = 0.2776, *p* = 0.0298). PBP1 showed a borderline inverse association (R^2^ = 0.2836, *p* = 0.0500), whereas the placebo group showed no significant association. Solid lines indicate linear regression fits with 95% confidence intervals.

Meanwhile, correlation analysis between tryptophan–indole metabolism and zonulin (Figure 8) demonstrated positive associations between decreases in tryptophan and zonulin, and inverse associations between increases in indole and zonulin, with statistical significance observed in the PBP2 group. These findings suggest that enhanced microbial conversion of tryptophan into indole metabolites may be associated with reduced intestinal permeability. Indole and its derivatives have been reported to support intestinal barrier function through activation of host signaling pathways such as PXR and AhR, leading to increased expression of tight junction proteins and mucin production [48,49]. In addition, butyrate has been shown to suppress claudin-2 expression and reinforce epithelial barrier integrity [33]. Taken together, these findings provide supportive mechanistic evidence linking microbial metabolite production with epithelial barrier regulation and are consistent with the barrier-enhancing effects observed in the organoid model.

Regarding microbial diversity, the core phylogenetic structure remained relatively stable, consistent with the resilience of the adult gut microbiota. However, compositional shifts were observed at finer taxonomic levels, indicating targeted modulation rather than broad ecological disruption. At the genus and higher taxonomic levels, supplementation was associated with coordinated shifts in microbial composition. Both PBP1 and PBP2 groups showed increases in Lactobacillaceae-related taxa, including *Lactobacillus*, consistent with the administered probiotic strains, as well as increases in *Gordonibacter*, which has been associated with the metabolism of plant-derived polyphenols [47]. In addition, several taxa linked to carbohydrate fermentation and microbial metabolism—including *Prevotella*, *Eubacterium* group, and *Oscillibacter*—were increased in both intervention groups, which is consistent with the observed elevations in SCFA levels [10,11]. Members of the *Eubacterium* group contribute to butyrate production via the butyryl-CoA:acetate CoA-transferase pathway, while *Prevotella* species support SCFA production through carbohydrate fermentation leading to acetate generation [10]. Furthermore, *Oscillibacter* has been reported to be reduced in obese individuals and to exhibit positive correlations with butyrate and isobutyrate levels, along with negative associations with body weight, BMI, body fat percentage, and HOMA-IR [56]. These findings may indicate that the increased abundance of *Prevotella*, *Eubacterium* group, and *Oscillibacter* observed following PBP1 and PBP2 supplementation may also be linked to enhanced SCFA production. PBP2-specific increases were observed in Muribaculaceae, Bacillales, and Bacillaceae, suggesting a potential functional enrichment of the gut microbial ecosystem. In addition, Muribaculaceae are known to metabolize both dietary and host-derived polysaccharides and engage in cooperative interactions with probiotic genera such as *Bifidobacterium* and *Lactobacillus* [57], which may explain their selective enrichment in the PBP2 group. The coordinated increase in SCFA-associated taxa across both intervention groups, along with PBP2-specific enrichment of functionally relevant microbes, supports the possibility that the observed microbiome shifts may be functionally aligned with enhanced fermentative metabolism and metabolite production, rather than representing purely compositional alterations. These compositional changes are consistent with the observed increases in fecal butyrate levels and reductions in zonulin, suggesting a potential link between microbiome modulation and improved intestinal barrier function.

This study has several limitations. First, the relatively small sample size may limit generalizability. In addition, the sample size calculation was based on fecal butyrate concentration, which was selected as a key microbiome-related endpoint. Consequently, the study may not have been sufficiently powered to detect smaller effects in other exploratory outcomes, including zonulin and tryptophan-related metabolites. Second, the 8-week intervention period may not capture long-term effects. Third, fecal analyses were conducted at limited time points, which may not fully reflect temporal dynamics of the microbiome and metabolome. Fourth, microbial functional activity was inferred indirectly from metabolite changes rather than directly measured, and several analyses should therefore be interpreted as exploratory. Future studies integrating multi-omics approaches, larger sample sizes, and predefined powered endpoints are warranted to confirm the observed findings and clarify their clinical significance. In conclusion, supplementation with PBP1 and PBP2, composed of probiotics and phytonutrients, improved bowel function and was associated with alterations in gut microbial composition and metabolic activity. In particular, the increase in SCFAs, including butyrate, and the shift in tryptophan–indole metabolism was associated with improvements in markers related to intestinal epithelial barrier function. These findings suggest that microbiota-derived metabolites may play a key role in maintaining gut function and barrier homeostasis. Overall, this study highlights the potential of probiotic–phytonutrient formulations to improve gut health through modulation of microbial metabolism and supports the need for further research across diverse formulations and populations.

## 5. Conclusions

In this randomized, double-blind, placebo-controlled trial, supplementation with PBP1 and PBP2 improved bowel function and was associated with improvements in stool normalization, increased fecal SCFA levels, particularly butyrate, and alterations in tryptophan metabolism. These changes were accompanied by reductions in zonulin, a marker associated with intestinal permeability. Collectively, these findings suggest that probiotic–phytonutrient supplementation may influence gut microbial activity and gut barrier-related biomarkers through microbiome-mediated metabolic modulation. However, given the exploratory nature of the study and the reliance on surrogate outcome measures, further studies are needed to confirm these findings and determine their clinical significance.

## Figures and Tables

**Table 1:** Baseline demographics and clinical characteristics.

Summary Statistics			PBP1 (*n* = 17)	PBP2 (*n* = 20)	Placebo (*n* = 17)	*p*-Value		
						*p* ^a^	*p* ^b^	*p* ^c^
Age		Mean (SD)	40.18 (10.34)	41.10 (9.53)	39.47 (8.80)	0.8316	0.5948	0.8740
Sex	Female	% (*n*)	58.82 (10)	60.00 (12)	47.06 (8)	0.4920	0.4312	0.6940
	Male	% (*n*)	41.18 (7)	40.00 (8)	52.94 (9)			
BMI (kg/m^2^)		Mean (SD)	24.23 (4.02)	23.36 (3.04)	22.85 (3.18)	0.2782	0.6264	0.5002
	BMI < 25.0	% (*n*)	47.06 (8)	70.00 (14)	70.59 (12)	0.1634	0.9689	0.2602
	25.0 ≤ BMI < 30.0	% (*n*)	52.94 (9)	30.00 (6)	29.41 (5)			
Stool consistency		Mean (SD)	4.12 (0.99)	4.25 (1.25)	4.06 (1.25)	0.8801	0.6723	0.8785
	Constipation (BSFS type 1–2)	% (*n*)	11.76 (2)	5.00 (1)	11.76 (2)	1.0000	0.6305	0.7446
	Normal (BSFS type 3–5)	% (*n*)	82.35 (14)	75.00 (15)	76.47 (13)			
	Diarrhea (BSFS type 6–7)	% (*n*)	5.88 (1)	20.00 (4)	11.76 (2)			
Fecal Biomarkers								
	SCFAs (μg/g)	Mean (SD)	67.94 (20.94)	68.01 (18.42)	82.33 (26.95)	0.0644	0.0558	0.0974
	Butyrate (μg/g)	Mean (SD)	9.47 (4.90)	8.82 (4.05)	11.58 (5.79)	0.2160	0.0950	0.2242
	Zonulin (ng/mL)	Mean (SD)	299.88 (187.75)	246.89 (175.54)	240.99 (181.47)	0.3481	0.9218	0.5777

*p*-values were calculated using independent *t*-tests or one-way ANOVA for continuous variables, and chi-square test or Fisher’s exact test for categorical variables. *p* ^a^: PBP1 vs. Placebo; *p* ^b^: PBP2 vs. Placebo; *p* ^c^: overall group difference. BMI: Body Mass Index; BSFS: Bristol stool form scale; SCFAs: Short-Chain Fatty Acids.

**Table 2:** Stool pattern based on the Bristol Stool Scale (7-day diary).

Group Week		Constipation (BSFS Type 1–2)			Normal (BSFS Type 3–5)			Diarrhea (BSFS Type 6–7)		
			*p* ^†^	*p* ^‡^		*p* ^†^	*p* ^‡^		*p* ^†^	*p* ^‡^
PBP1	0	9.51 ± 12.72			74.10 ± 22.80			16.39 ± 17.55		
	4	6.71 ± 14.43	0.4904	0.3727	76.56 ± 26.45	0.6830	0.1432	16.73 ± 21.43	0.9275	0.2491
	8	2.57 ± 6.09	0.0130	0.0532	88.07 ± 17.45	0.0005	0.0151	9.36 ± 13.86	0.0054	0.0762
PBP2	0	8.42 ± 14.05			67.44 ± 29.06			24.14 ± 25.83		
	4	5.00 ± 15.39	0.5117	0.1787	82.94 ± 23.19	0.0438	0.0360	12.06 ± 17.21	0.0231	0.0188
	8	4.07 ± 7.62	0.1444	0.2518	79.67 ± 20.74	0.0304	0.0226	16.26 ± 18.82	0.1128	0.3284
Placebo	0	13.01 ± 17.41			70.39 ± 25.57			16.61 ± 27.23		
	4	15.82 ± 18.27	0.2402		67.01 ± 22.19	0.5127		17.18 ± 20.80	0.8893	
	8	16.42 ± 24.20	0.4563		67.08 ± 27.59	0.5321		16.50 ± 20.71	0.9729	

*p* ^†^-values represent within-group comparisons versus baseline at Week 4 and 8, analyzed using McNemar’s test. *p* ^‡^-values represent between-group differences in change from baseline at Week 4 and 8, analyzed using the chi-square test or Fisher’s exact test, as appropriate.

**Table 3:** Changes in gastrointestinal symptoms, and bowel habits during the intervention period.

Contents	Week	PBP1	PBP2	Placebo	*p* ^†^	*p* ^‡^
Bowel movement frequency	0	3.88 ± 1.17	3.50 ± 0.95	3.41 ± 0.87		
	4	4.12 ± 0.93	3.20 ± 0.95	3.94 ± 0.90	0.1424	0.0015 **
	8	4.12 ± 0.86	3.50 ± 0.83	3.71 ± 0.99	0.3942	0.2381
Defecation time	0	1.71 ± 0.47	1.70 ± 0.73	1.71 ± 0.59		
	4	1.65 ± 0.61	1.75 ± 0.64	1.76 ± 0.66	0.4363	1.0000
	8	1.59 ± 0.51	1.60 ± 0.60	1.71 ± 0.69	0.5004	0.7456
Abdominal pain	0	1.06 ± 1.98	1.35 ± 2.50	0.71 ± 1.26		
	4	1.35 ± 2.32	1.35 ± 2.30	1.47 ± 2.24	0.3055	0.1255
	8	1.06 ± 2.22	0.95 ± 1.67	1.06 ± 1.75	0.0896	0.1483
Abdominal bloating	0	2.41 ± 2.60	2.15 ± 2.48	3.53 ± 2.62		
	4	2.35 ± 2.76	2.80 ± 2.89	2.41 ± 2.60	0.3329	0.0845
	8	2.35 ± 2.74	1.60 ± 2.04	2.12 ± 1.96	0.2434	0.5155
Incomplete evacuation sensation	0	2.47 ± 2.55	1.75 ± 2.67	1.53 ± 2.10		
	4	1.76 ± 2.02	1.85 ± 2.87	1.59 ± 1.91	0.3849	0.6542
	8	1.18 ± 1.78	1.15 ± 1.81	0.94 ± 1.68	0.2840	0.8348
Postprandial bowel urgency	0	2.06 ± 2.59	1.05 ± 1.70	1.06 ± 1.34		
	4	1.24 ± 1.95	0.95 ± 1.54	1.71 ± 2.05	0.0066 **	0.0739
	8	1.06 ± 1.75	0.55 ± 1.15	1.24 ± 1.71	0.0029 **	0.1419

Values are presented as mean ± SD. Between-group comparisons were performed using the Mann–Whitney U test. *p* ^†^: Comparison between PBP1 vs. placebo; *p* ^‡^: Comparison between PBP2 vs. placebo. ** *p* < 0.01.

## Data Availability

Data sharing is not applicable to this article due to ethical policy.

## Supplementary Materials

The following supporting information can be downloaded at: https://www.mdpi.com/article/10.3390/nu18132085/s1, Supplementary Method; Table S1: Quantifier multiple reaction monitoring transition of tryptophan metabolism related metabolites; Table S2: Summary of adverse events reported during the intervention period; Figure S1: Individual changes in short-chain fatty acid (SCFA) levels following PBP1 and PBP2 supplementation; Figure S2: Individual short-chain fatty acid (SCFA) profiles following PBP1 and PBP2 supplementation; Figure S3: Individual changes in fecal zonulin levels following PBP1 and PBP2 supplementation; Figure S4: Changes in indole-derived tryptophan metabolites following PBP1 and PBP2 supplementation; Figure S5: Changes in alpha-diversity across taxonomic levels following PBP1 and PBP2 supplementation; Figure S6: Characterization of human intestinal organoid-derived monolayers; Figure S7: PBP2 preserves intestinal epithelial barrier function under inflammatory conditions; Figure S8: Effect of PBP2 on tight junction-related gene and protein expression in human intestinal organoid-derived monolayers.
