# Supplementary material for: Effects of Probiotic–Phytonutrient Blends on Defecation, Intestinal Barrier Function, and Gut Microbiota: A Randomized, Placebo-Controlled Trial
Source: Nutrients. 2026 Jun 25;18(13):2085. doi: 10.3390/nu18132085 (PMC13363449; doi:10.3390/nu18132085)
Supplement: Supplementary file 1 [file nutrients-18-02085-s001.zip › Supplementary Figure 1_R2.pdf]

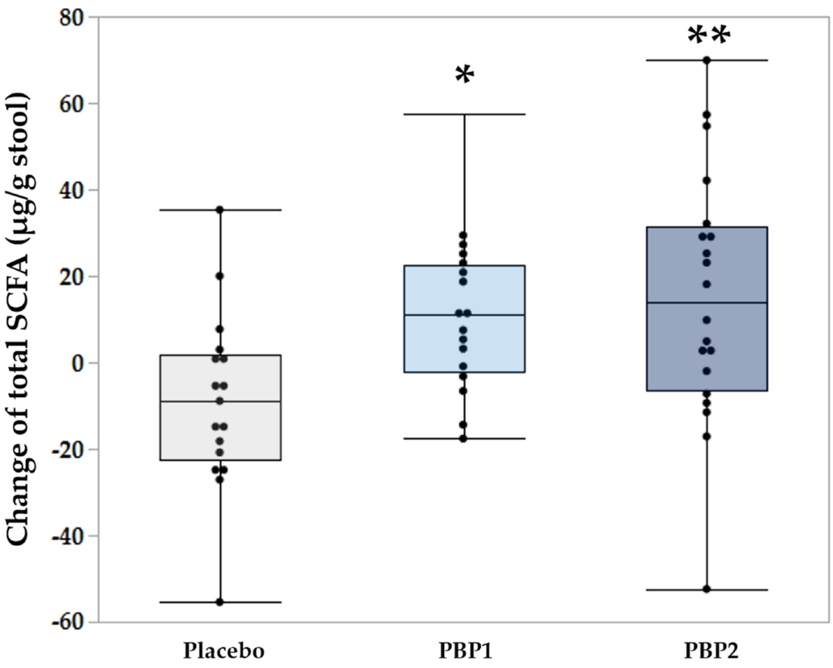

Supplementary Figure 1. Individual changes in short-chain fatty acid (SCFA) levels following PBP1 and PBP2 supplementation. Boxplots with individual data points represent changes from baseline to Week 8 in total SCFA levels in the placebo, PBP1, and PBP2 groups. Box plots display median and interquartile range, with individual data points overlaid. Significant increases in total SCFA levels were observed in the PBP1 and PBP2 groups compared with the placebo group. * $p < 0.05$, ** $p < 0.01$ vs. placebo.
