# Supplementary material for: Effects of Probiotic–Phytonutrient Blends on Defecation, Intestinal Barrier Function, and Gut Microbiota: A Randomized, Placebo-Controlled Trial
Source: Nutrients. 2026 Jun 25;18(13):2085. doi: 10.3390/nu18132085 (PMC13363449; doi:10.3390/nu18132085)
Supplement: Supplementary file 1 [file nutrients-18-02085-s001.zip › Supplementary Figure 2_R2.pdf]

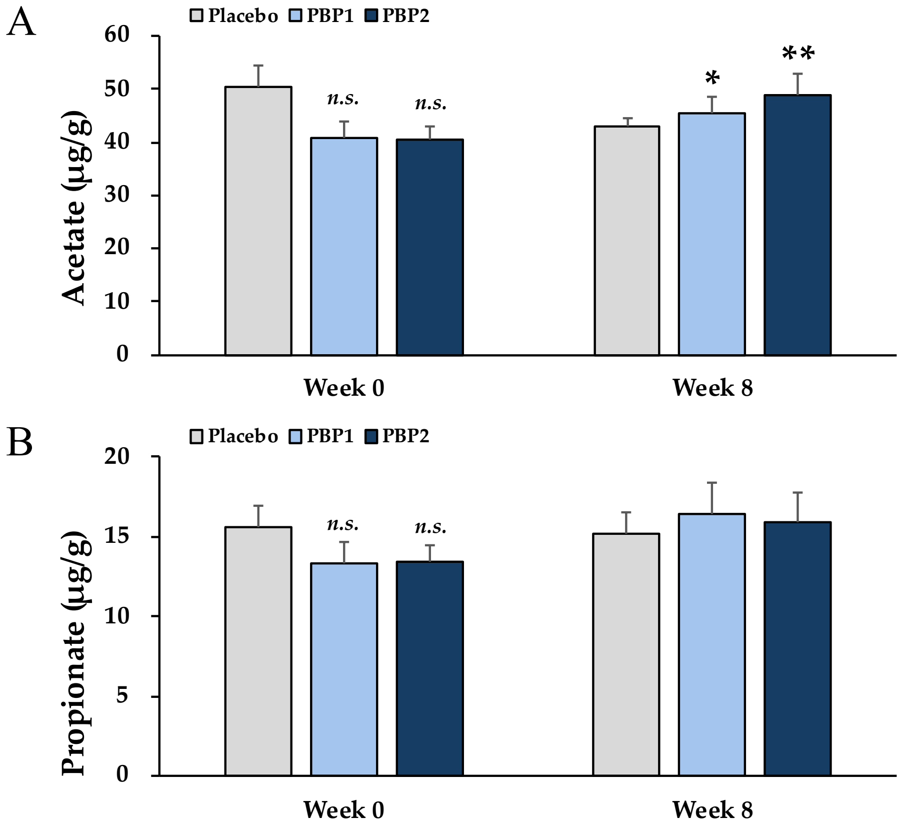

Supplementary Figure 2. Individual short-chain fatty acid (SCFA) profiles following PBP1 and PBP2 supplementation. Fecal acetate (A) and propionate (B) concentrations at baseline (Week 0) and Week 8 are shown for the placebo, PBP1, and PBP2 groups. Bars represent mean ± standard error (SE). No significant differences were observed among groups at baseline. Similar to butyrate, acetate levels showed significant increases following PBP1 and/or PBP2 supplementation, whereas propionate levels exhibited only increasing trends without statistical significance. Although absolute values are displayed, statistical comparisons were performed using change values from baseline. * $p < 0.05$, ** $p < 0.01$ vs. placebo; n.s., not significant.
