# Supplementary material for: Effects of Probiotic–Phytonutrient Blends on Defecation, Intestinal Barrier Function, and Gut Microbiota: A Randomized, Placebo-Controlled Trial
Source: Nutrients. 2026 Jun 25;18(13):2085. doi: 10.3390/nu18132085 (PMC13363449; doi:10.3390/nu18132085)
Supplement: Supplementary file 1 [file nutrients-18-02085-s001.zip › Supplementary Figure 3_R2.pdf]

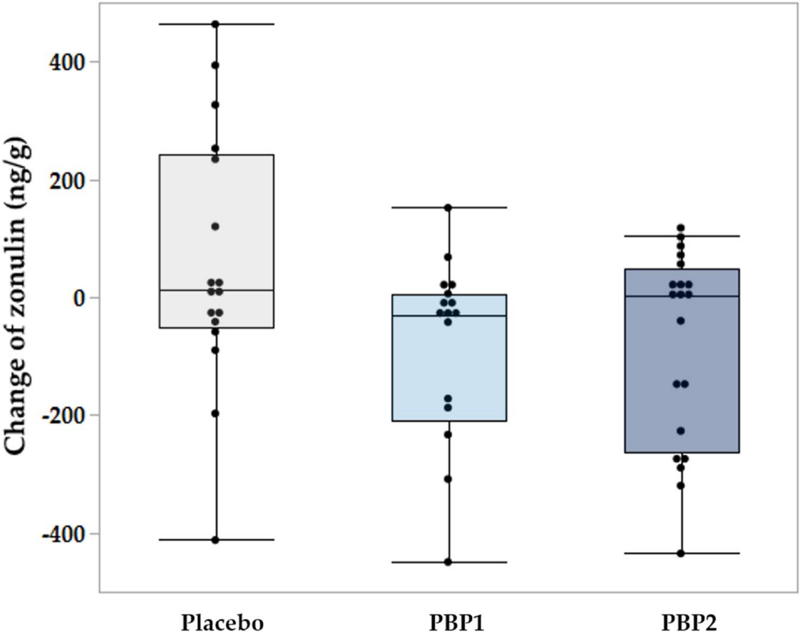

Supplementary Figure 3. Individual changes in fecal zonulin levels following PBP1 and PBP2 supplementation. Boxplots with individual data points represent changes from baseline to Week 8 (Week 8 − Week 0) in fecal zonulin levels in the placebo, PBP1, and PBP2 groups. Box plots display median and interquartile range, with individual data points overlaid.
