# Supplementary material for: Effects of Probiotic–Phytonutrient Blends on Defecation, Intestinal Barrier Function, and Gut Microbiota: A Randomized, Placebo-Controlled Trial
Source: Nutrients. 2026 Jun 25;18(13):2085. doi: 10.3390/nu18132085 (PMC13363449; doi:10.3390/nu18132085)
Supplement: Supplementary file 1 [file nutrients-18-02085-s001.zip › Supplementary Figure 4_R2.pdf]

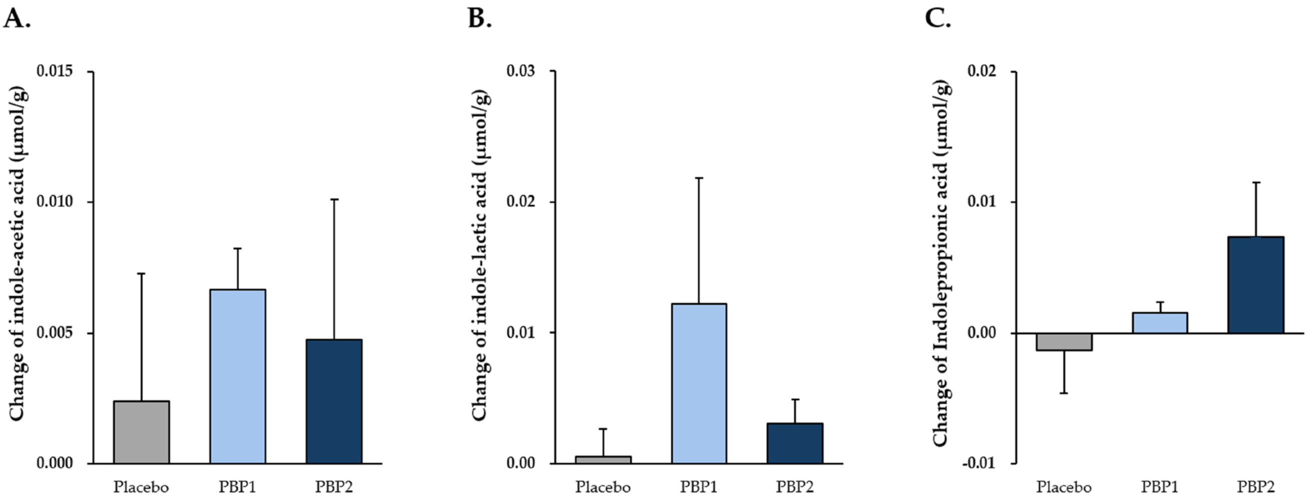

Supplementary Figure 4. Changes in indole-derived tryptophan metabolites following PBP1 and PBP2 supplementation. Changes from baseline to Week 8 (Δ, Week 8 − Week 0) in fecal indole-3-acetic acid (IAA) (A), indole-lactic acid (ILA) (B), and indolepropionic acid (IPA) (C) levels are shown for the placebo, PBP1, and PBP2 groups. Bars represent mean ± standard error (SE). Although no statistically significant differences were observed between groups, IAA, ILA, and IPA levels exhibited consistent upward trends in the PBP1 and PBP2 groups compared with the placebo group.
