# Supplementary material for: Effects of Probiotic–Phytonutrient Blends on Defecation, Intestinal Barrier Function, and Gut Microbiota: A Randomized, Placebo-Controlled Trial
Source: Nutrients. 2026 Jun 25;18(13):2085. doi: 10.3390/nu18132085 (PMC13363449; doi:10.3390/nu18132085)
Supplement: Supplementary file 1 [file nutrients-18-02085-s001.zip › Supplementary Figure5_R2.pdf]

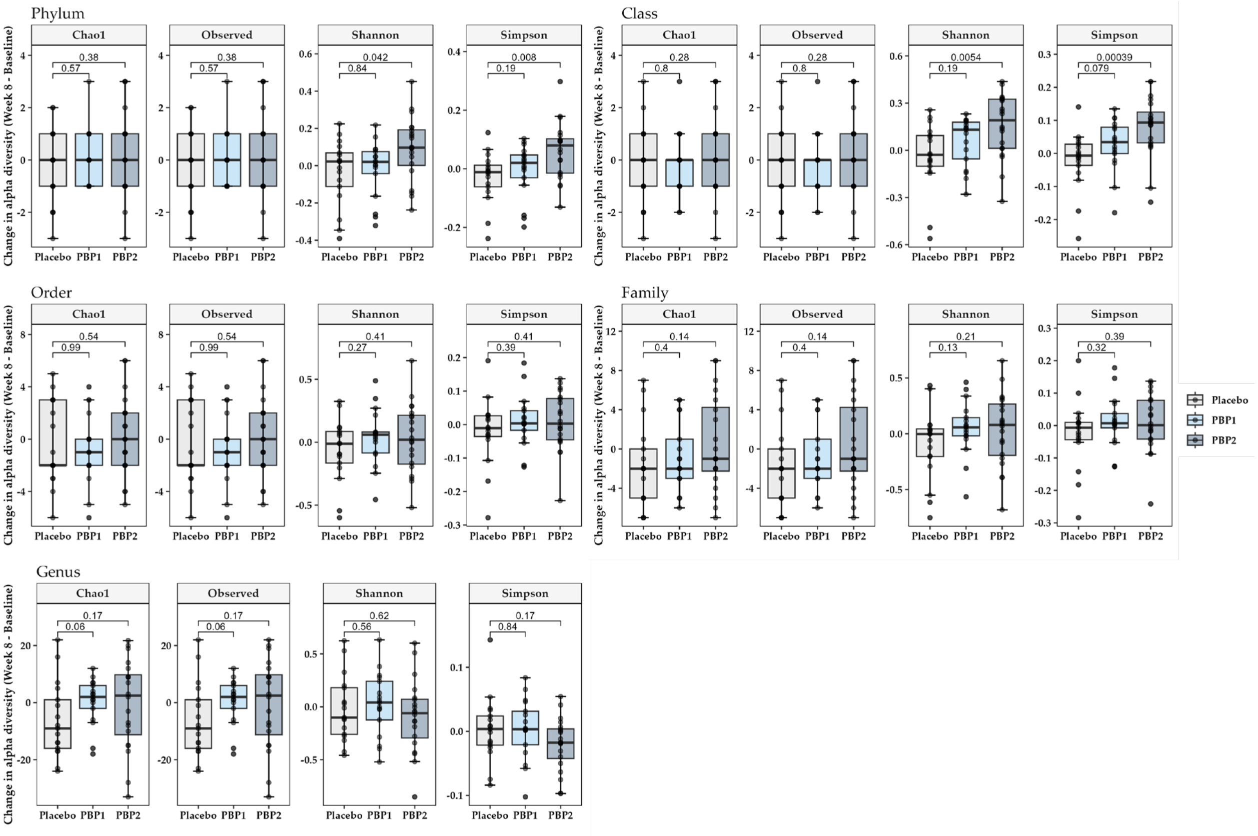

Supplementary Figure 5. Changes in alpha-diversity across taxonomic levels following PBP1 and PBP2 supplementation. Changes in alpha-diversity indices (Chao1, observed richness, Shannon, and Simpson) from baseline to Week 8 were evaluated at the phylum, class, order, family, and genus levels across the placebo, PBP1, and PBP2 groups. Statistical comparisons were performed using the Wilcoxon rank-sum test. Chao1 and observed richness analyses showed minimal changes at the phylum and class levels, whereas greater divergence was observed at the order, family, and genus levels.
