# Supplementary material for: Effects of Probiotic–Phytonutrient Blends on Defecation, Intestinal Barrier Function, and Gut Microbiota: A Randomized, Placebo-Controlled Trial
Source: Nutrients. 2026 Jun 25;18(13):2085. doi: 10.3390/nu18132085 (PMC13363449; doi:10.3390/nu18132085)
Supplement: Supplementary file 1 [file nutrients-18-02085-s001.zip › Supplementary Figure6_R2.pdf]

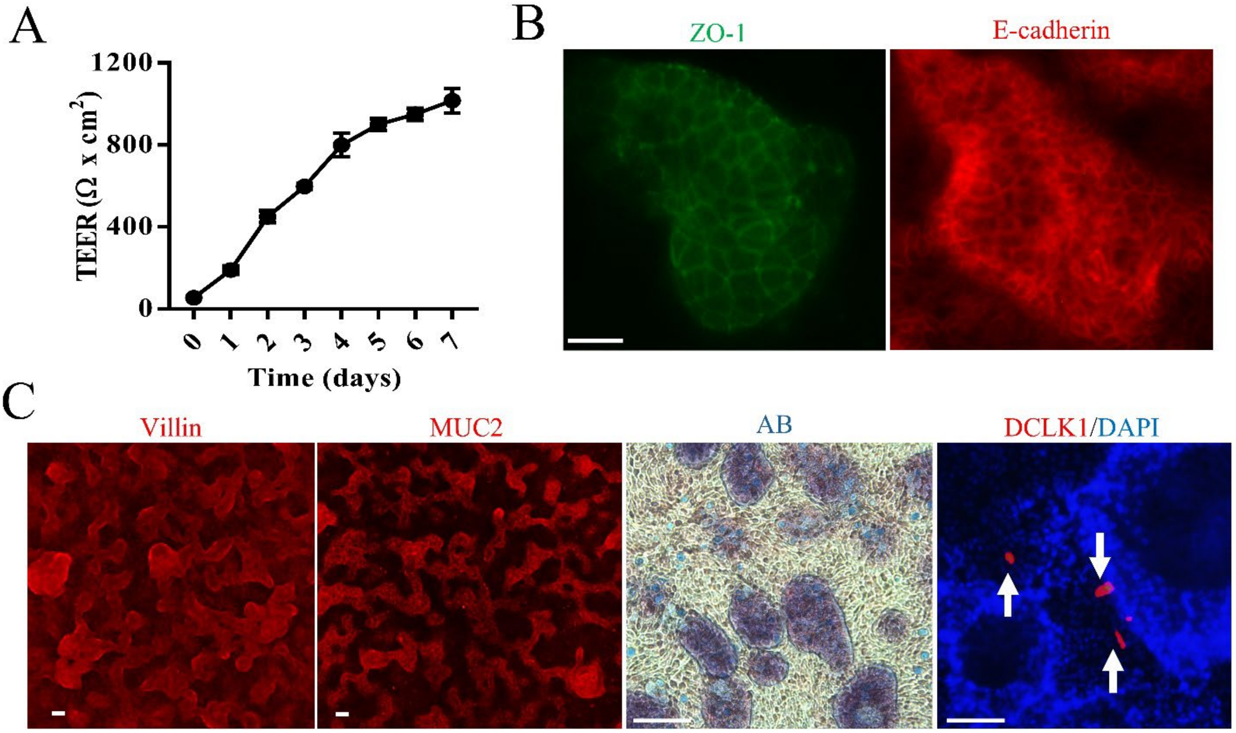


**Supplementary Figure 6. Characterization of human intestinal organoid-derived monolayers.** Single cells generated from human intestinal organoids were seeded onto 96-well Transwell inserts and differentiated for 7 days to establish epithelial monolayers. Barrier development and epithelial differentiation were assessed by TEER and marker staining. (A) TEER values measured over 7 days. (B) Representative immunofluorescence images of ZO-1 (green) and E-cadherin (red), indicating tight junction and adherens junction organization. Scale bars = 50 µm. (C) Representative images of epithelial differentiation markers: villin (red) for enterocytes, Alcian blue staining for acidic mucins, MUC2 (red) for goblet cells, and DCLK1 (red) for tuft cells. Nuclei were counterstained with DAPI where indicated. Scale bars = 100 µm. Data are presented as mean ± SEM.
