# Supplementary material for: Effects of Probiotic–Phytonutrient Blends on Defecation, Intestinal Barrier Function, and Gut Microbiota: A Randomized, Placebo-Controlled Trial
Source: Nutrients. 2026 Jun 25;18(13):2085. doi: 10.3390/nu18132085 (PMC13363449; doi:10.3390/nu18132085)
Supplement: Supplementary file 1 [file nutrients-18-02085-s001.zip › Supplementary Figure7_R2.pdf]

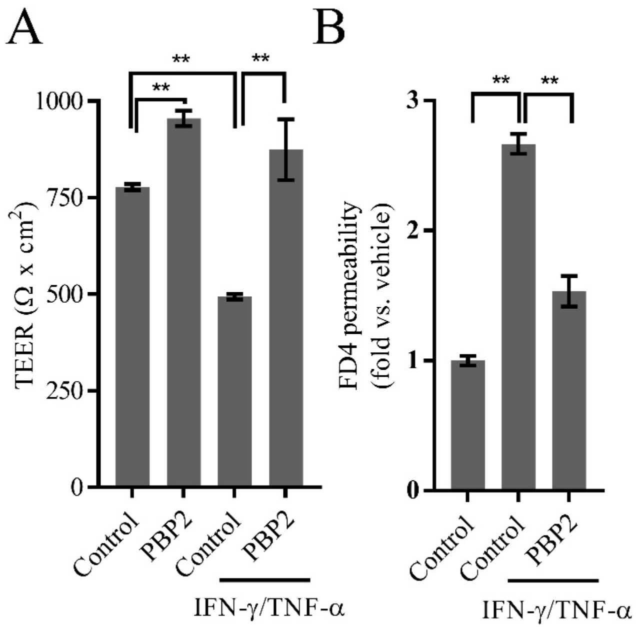

Supplementary Figure 7. PBP2 preserves intestinal epithelial barrier function under inflammatory conditions. Human intestinal organoid-derived monolayers were pretreated with PBP2-derived conditioned media for 24 h and then exposed to IFN-γ and TNF-α at 40 ng/mL each for 48 h. (A) TEER was measured as a functional readout of barrier integrity. (B) Paracellular permeability was assessed by quantifying basolateral flux of 4 kDa FITC-dextran (FD4). All data were generated using 96-well Transwell cultures. Data are presented as mean ± SEM; n = 6 per group. $^{**}p < 0.01$.
