# Supplementary material for: Effects of Probiotic–Phytonutrient Blends on Defecation, Intestinal Barrier Function, and Gut Microbiota: A Randomized, Placebo-Controlled Trial
Source: Nutrients. 2026 Jun 25;18(13):2085. doi: 10.3390/nu18132085 (PMC13363449; doi:10.3390/nu18132085)
Supplement: Supplementary file 1 [file nutrients-18-02085-s001.zip › Supplementary Figure8_R2.pdf]

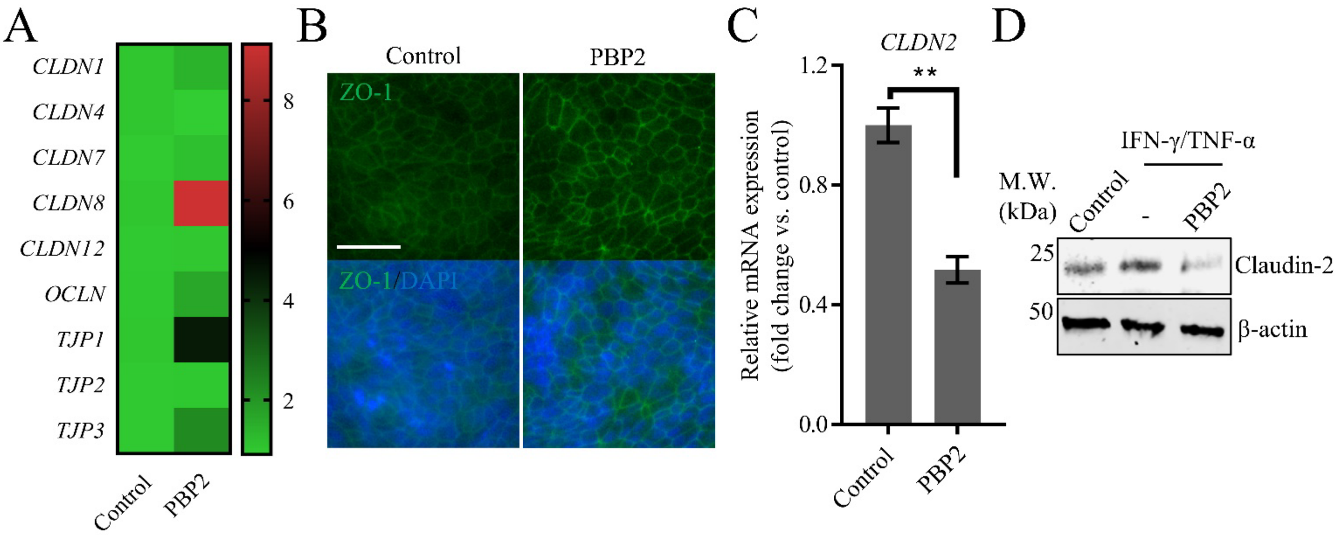

Supplementary Figure 8. Effect of PBP2 on tight junction-related gene and protein expression in human intestinal organoid-derived monolayers. Monolayers were treated with PBP2-derived conditioned media, and tight junction-associated markers were analyzed. (A) Heatmap of selected tight junction-related transcripts under basal, non-inflammatory conditions. (B) Representative ZO-1 immunofluorescence images from control and PBP2-treated monolayers under basal conditions. Scale bar = 50 µm. (C) Relative *CLDN2* mRNA expression in control and PBP2-treated monolayers under basal conditions, normalized to GAPDH using the $2^{\wedge-\Delta\Delta Ct}$ method. (D) Western blot analysis of claudin-2 protein expression after IFN-γ/TNF-α challenge with or without PBP2 pretreatment. β-actin was used as a loading control. Data are presented as mean ± SEM from three independent experiments. $**p < 0.01$.
