# Supplementary material for: Effects of Probiotic–Phytonutrient Blends on Defecation, Intestinal Barrier Function, and Gut Microbiota: A Randomized, Placebo-Controlled Trial
Source: Nutrients. 2026 Jun 25;18(13):2085. doi: 10.3390/nu18132085 (PMC13363449; doi:10.3390/nu18132085)
Supplement: Supplementary file 1 [file nutrients-18-02085-s001.zip › Supplementary Methods_R2.pdf]

2. Materials and Methods

2.12. Organoid-derived monolayer culture

Human small intestinal organoids were obtained from Dr. Jason's laboratory at the University of Michigan. Organoids were expanded as Matrigel dome cultures using growth factor-reduced Matrigel (Cat#356231; Corning) and IntestiCult™ Organoid Growth Medium (OGM). The complete OGM consisted of IntestiCult™ OGM Human Basal Medium (Cat#100-0190, STEMCELL Technologies) and IntestiCult™ Organoid Supplement (Cat#100-0191, STEMCELL Technologies), prepared according to the manufacturer's recommendations. Y-27632 (Cat#S1049; Selleck Chemicals) and Primocin (Cat#ant-pm-2; InvivoGen) were added where indicated. Organoids were passaged every 7–8 days by mechanical disruption followed by incubation in Gentle Cell Dissociation Reagent (Cat#100-0485, STEMCELL Technologies) for 5–10 min at room temperature. Pelleted fragments were washed briefly with DMEM/F-12 medium (Cat#36254, STEMCELL Technologies), resuspended in Matrigel, and overlaid with pre-warmed culture medium. Cultures were maintained at 37°C and 5% CO_2 in a humidified incubator, with medium replacement every 2–3 days. For monolayer generation, organoids were dissociated into single cells using a previously reported protocol with minor modifications [58]. Organoids were sequentially treated with Gentle Cell Dissociation Reagent and 0.05% Trypsin-EDTA (Cat#07910, STEMCELL Technologies). Trypsinization was stopped with DMEM/F-12 medium containing 10% fetal bovine serum (FBS). The resulting single-cell suspension was seeded onto Matrigel-coated HTS Transwell® 96-well inserts (0.4 μm pore size, Cat#7369, Corning). Cells were maintained in IntestiCult™ Organoid Differentiation Medium (ODM; Cat#100-0214, STEMCELL Technologies), and medium was replaced every other day. Barrier development was followed by transepithelial electrical resistance (TEER) measurements.

2.13. TEER measurements

TEER was measured with the EVOM™ Auto TEER Measurement System (EVA-MT-03-01, World Precision Instruments). Final TEER values were obtained by multiplying the raw resistance readings by the membrane surface area of the Transwell insert.

2.14. Permeability assay

Paracellular permeability was measured using 4 kDa FITC-dextran (Cat#46944-500MG-F, Sigma-Aldrich, USA). After PBS washing, 0.25 mM FITC-dextran was placed in the apical chamber, and ODM was added to the basolateral chamber. Monolayers were incubated for 1 h at 37°C with 5% CO_2, after which basolateral medium was collected. Fluorescence intensity was quantified using a SpectraMax iD5 plate reader (Molecular Devices). Values were corrected for ODM autofluorescence and normalized to the vehicle control.

2.15. Sample and cytokine treatment

Monolayers were exposed to test samples diluted 1:100 in ODM for 24 h before cytokine challenge. Cytotoxicity was evaluated with the CellTiter-Glo® 3D Cell Viability Assay (Cat#G9682, Promega) following the manufacturer's protocol. After pretreatment, inflammatory barrier disruption was induced by adding recombinant human IFN-γ (Cat#300-02-20UG, Lot#091927; PeproTech) and recombinant human TNF-α (Cat#210-TA, Lot#DDHB0421121; R&D Systems), each at 40 ng/mL, for an additional 48 h. Monolayers were then processed for barrier function assays and molecular analyses.

2.16. Immunofluorescence microscopy

Monolayers were fixed in 4% paraformaldehyde for 10 min and permeabilized with 0.3% Triton X-100 in PBS for 20 min at room temperature. Cells were blocked for 1 h with 5% BSA in PBS. Primary antibodies against ZO-1 (Cat#33-9100, Lot#WL337900; Thermo Fisher Scientific), claudin-2 (Cat#51-6100, Lot#YG373730A; Invitrogen), villin (Cat#sc-58897, Lot#11624; Santa Cruz Biotechnology), MUC2 (Cat#MA5-12345, Lot#XC3543824; Invitrogen), and E-cadherin (Cat#3195, Lot#15; Cell Signaling Technology) were prepared in 2.5% BSA in PBS and applied overnight at 4°C. Following three PBS washes, samples were incubated for 1 h at room temperature in the dark with Alexa Fluor-conjugated secondary antibodies: Alexa Fluor™ 594 goat anti-mouse IgG (H+L) (Cat#A11037, Lot#3174388, Thermo Fisher Scientific), Alexa Fluor™ Plus 488 goat anti-rabbit IgG (H+L) (Cat#A32731, Lot#XA339480, Thermo Fisher Scientific), Alexa Fluor™ Plus 594 goat anti-mouse IgG (H+L) (Cat#A32742, Lot#WG328516), or Alexa Fluor™ Plus 488 goat anti-mouse IgG (H+L) (Cat#A32723, Lot#ZB388196, Thermo Fisher Scientific). Nuclei were counterstained with DAPI, and images were captured using a Leica DMI6000B microscope.

2.17. Analysis of tight junction–associated genes

Total RNA was extracted with the RNeasy Kit (QIAGEN), and cDNA was generated using the iScript cDNA Synthesis Kit (Bio-Rad) according to the manufacturers' protocols. Quantitative real-time PCR was performed on a Bio-Rad CFX96 Real-Time PCR Detection System. Tight junction-associated transcripts were analyzed using the RT^2 Profiler™ PCR Array Human Tight Junctions (Cat#330231, QIAGEN) with RT^2 SYBR® Green qPCR Mastermix (Cat#330502, QIAGEN). Melting curve analysis was used to verify PCR specificity, and GAPDH served as the internal reference gene.

2.18. Immunoblotting

For immunoblotting, treated samples were lysed in RIPA buffer containing protease inhibitors. Protein concentrations were measured using Pierce™ Dilution-Free™ BSA Protein Standards (Cat#186882, Lot#AB397429; Thermo Fisher Scientific). Equal protein amounts were combined with Bio-Rad sample buffer, heat-denatured, separated on Mini-PROTEAN TGX 4–20% precast gels (Cat#4561094, Bio-Rad), and transferred to low-fluorescence PVDF membranes (Cat#1620260, Bio-Rad) using the Trans-Blot Turbo Transfer System (Bio-Rad). Membranes were blocked with Intercept® TBS Blocking Buffer (Cat#927-60001, LI-COR Biosciences) for 1.5 h at room temperature and incubated with primary antibodies overnight at 4°C. After TBST washing, membranes were incubated with IRDye® secondary antibodies, including IRDye® 800CW goat anti-rabbit IgG (Cat#925-32211, Lot#D30110-01) or IRDye® 680RD goat anti-mouse IgG (Cat#925-68070, Lot#D302074; LI-COR Biosciences). Fluorescent signals were acquired using the Odyssey CLx Imaging System (LI-COR Biosciences) and quantified with Image Studio software.

2.19. Statistical analysis for in vitro experiments

Data were analyzed using GraphPad Prism 7 software (GraphPad Software, USA). Group differences were assessed by one-way analysis of variance (ANOVA). Statistical significance was defined as $p < 0.01$, and data are shown as mean ± SEM.
